# Cellular uptake, tissue penetration, biodistribution, and biosafety of threose nucleic acids: Assessing in vitro and in vivo delivery

**DOI:** 10.1016/j.mtbio.2022.100299

**Published:** 2022-05-18

**Authors:** Fei Wang, Ling Sum Liu, Pan Li, Cia Hin Lau, Hoi Man Leung, Y Rebecca Chin, Chung Tin, Pik Kwan Lo

**Affiliations:** a Department of Chemistry, City University of Hong Kong, Tat Chee Avenue, Kowloon Tong, Hong Kong SAR, China; b Department of Biomedical Engineering, City University of Hong Kong, Tat Chee Avenue, Kowloon Tong, Hong Kong SAR, China; c Tung Biomedical Sciences Centre, Department of Biomedical Sciences, City University of Hong Kong, Tat Chee Avenue, Kowloon Tong, Hong Kong SAR, China; d Key Laboratory of Biochip Technology, Biotech and Health Care, Shenzhen Research Institute of City University of Hong Kong, Shenzhen, 518057, China

**Keywords:** TNA, Tissue penetration, Pharmacokinetics, Biodistribution, Biosafety

## Abstract

Compared with siRNAs or other antisense oligonucleotides (ASOs), the chemical simplicity, DNA/RNA binding capability, folding ability of tertiary structure, and excellent physiological stability of threose nucleic acid (TNA) motivate scientists to explore it as a novel molecular tool in biomedical applications. Although ASOs reach the target cells/tumors, insufficient tissue penetration and distribution of ASOs result in poor therapeutic efficacy. Therefore, the study of the time course of drug absorption, biodistribution, metabolism, and excretion is of significantly importance. In this work, the pharmacokinetics and biosafety of TNAs in living organisms are investigated. We found that synthetic TNAs exhibited excellent biological stability, low cytotoxicity, and substantial uptake in living cells without transfection. Using U87 three-dimensional (3D) multicellular spheroids to mimic the *in vivo* tumor microenvironment, TNAs showed their ability to penetrate efficiently throughout the whole multicellular spheroid as a function of incubation time and concentration when the size of the spheroid is relatively small. Additionally, TNAs could be safely administrated into Balb/c mice and most of them distributed in the kidneys where they supposed to excrete from the body through the renal filtration system. We found that accumulation of TNAs in kidneys induced no pathological changes, and no acute structural and functional damage in renal systems. The favourable biocompatibility of TNA makes it attractive as a safe and effective nucleic acid-based therapeutic agent for practical biological applications.

## Graphical abstract

**Figure:**
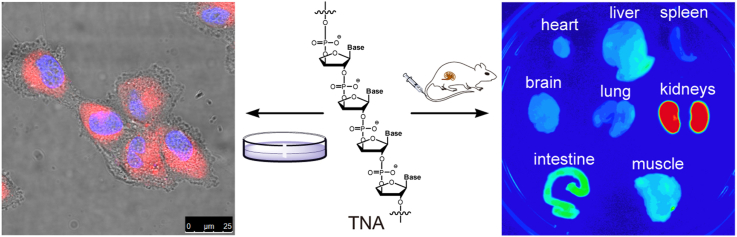

## 1 Introduction

The development of nucleic acid research offers an opportunity for the use of nucleic acid-based molecules for various biomedical applications, especially in disease therapeutics and diagnostics. For example, natural oligonucleotides including antisense oligonucleotides (ASOs), small interference RNAs (siRNAs), and ribozymes have shown great promise in modulating specific gene expression in a highly selective manner for cancer and other disease therapies [1,2]. However, natural DNA/RNAs exhibit poor target binding affinity, and therefore high doses are needed, leading to some side effects. Additionally, their acute enzymatic degradation and poor cellular uptake give rise to limited applications in clinical areas [3,4]. Viral gene-delivery vectors such as adenoviruses, retroviruses, or lentiviruses are commonly used for gene transfer *in vivo* but the application of this viral vector is hindered by severe immunological responses, high production cost, and low transgene packaging capacity [[5], [6], [7]]. Non-viral delivery systems involving the use of nanocarriers prepared from natural or synthetic compounds including polymers, cationic lipids, inorganic nanoparticles, quantum dots, and nucleic acid nanostructures have been developed as alternatives [[8], [9], [10]]. These nanoparticle-based carriers assist in promoting intracellular delivery, avoiding endonuclease degradation, and achieving the stimuli-responsive release of nucleic acids. Nevertheless, the nonspecific toxicity, insufficient transport to the targeted cells and tissues, and unsatisfying manufacture and quality control hinder the *in vivo* and clinical application of non-viral nanocarriers [2,11].

To improve the nuclease resistance, duplex-forming ability, and pharmacokinetic properties, scientists also put great effort into the chemical modification of the phosphodiester backbone, sugar moiety, or nucleobases of natural nucleic acids [12,13]. So far, a number of modified ASO analogues such as phosphorothioate oligonucleotides, peptide nucleic acids (PNAs), locked nucleic acids (LNAs), phosphorodiamidate morpholino oligomers, etc. show potentials as promising therapeutic agents to inhibit target gene expression by steric blocking [4,9,14]. However, they do suffer from poor cellular uptake, tendency to self-aggregate in aqueous media, weak binding affinity to natural oligonucleotides, and high toxicity for therapeutic applications [[15], [16], [17]]. Although the advent of a number of clinical trials in cancer and other disease therapies, the number of antisense drugs approved by the United States Food and Drug Administration is highly limited [18]. This results in slow progress in the development of nucleic acid-based therapeutics.

**Fig. 1:**
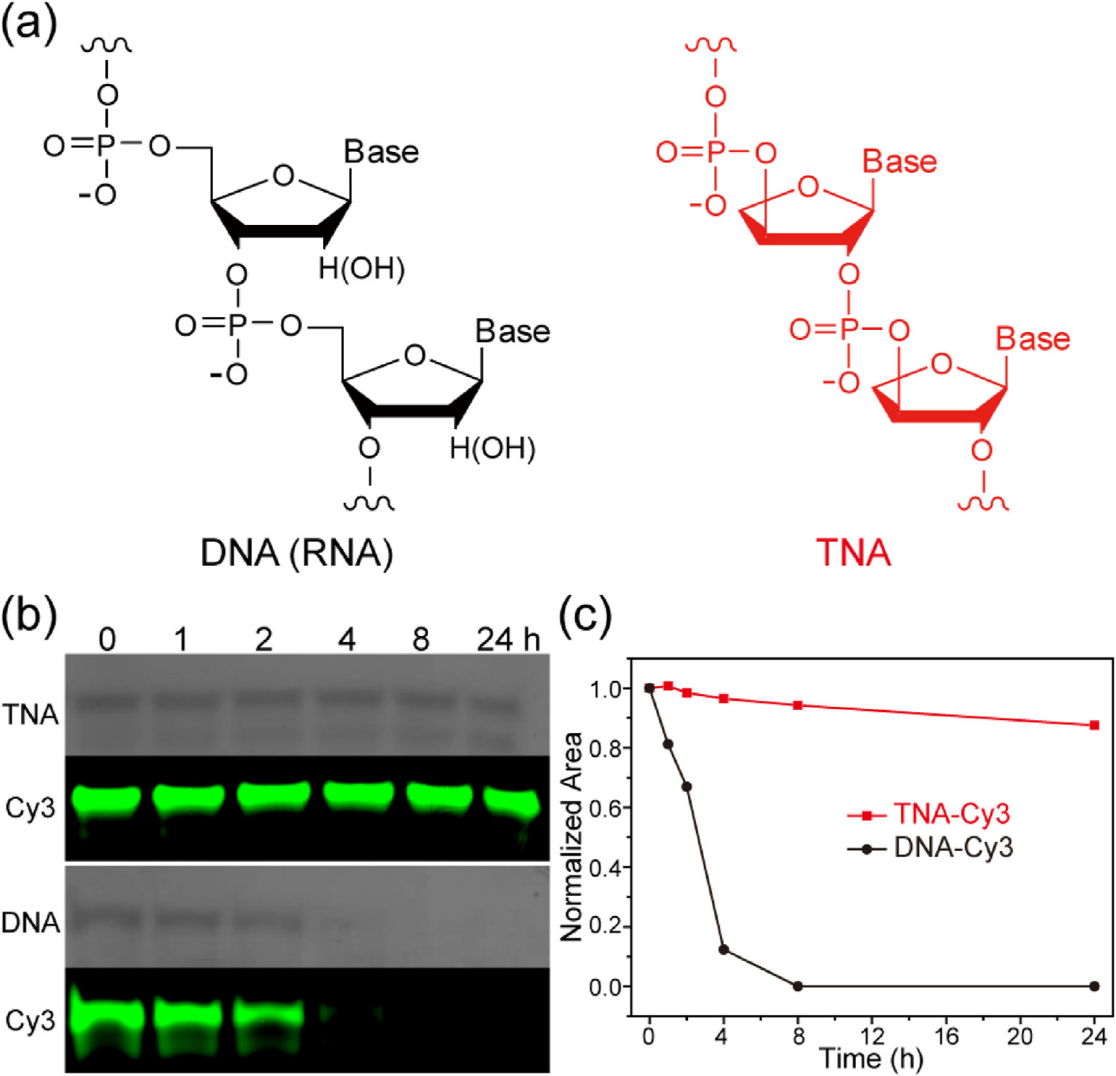
Chemical structures and physiological stability of DNA and TNA. (a) The chemical structures of DNA (RNA) and TNA oligonucleotides. (b) Denaturing PAGE analysis and fluorescence scanning of TNA-Cy3 and corresponding DNA-Cy3 strands after incubation with 10% FBS for designated time intervals (0, 1, 2, 4, 8, and 24 ​h). (c) Normalized intensity of TNA and DNA strands based on the denaturing PAGE results.

Recently, synthetic *α*-*l*-threose nucleic acid (TNA) which is composed of a backbone repeating of unnatural 4-carbon threose sugar with phosphodiester linkages taking place at the 2′- and 3′- vicinal positions of the threofuranose ring (Fig. 1a), has been considered as a possible RNA progenitor [19,20]. TNAs can form stable antiparallel duplexes with complementary sequences of DNA, RNA, and itself even though the sugar-phosphate backbone is one atom shorter than natural nucleic acids [20]. In addition, Chaput et al. showed that TNAs remain undigested after incubation in 50% human serum or human liver microsomes for 7 days and are highly resistant to a strong degradative snake venom phosphodiesterase (SVPDE). Furthermore, TNAs can protect internal DNA residues and shield complementary RNA strands from nuclease digestion [21]. The chemical simplicity, DNA/RNA binding capability, tertiary structure folding ability, and excellent physiological stability of TNAs motivate scientists to explore TNA as novel molecular tools in the area of biotechnology applications. So far, various biologically stable TNA aptamers targeting small molecules and large proteins have been selected by *in vitro* selection [22,23]. In particular, Yu and coworkers isolated biostable TNA aptamers that specifically target PD-L1 protein and demonstrated their potential *in vivo* anticancer immunotherapeutic efficacy [24]. Recently, our group found that sequence-controlled TNAs can be used in carrier-free condition for target gene suppression and *in vivo* antisense cancer therapy with no adverse toxicity [25,26]. Subsequently, Chaput et al. demonstrated that cytosine-phosphate-guanine (CpG) oligonucleotide sequence composed entirely of TNA units leads to the activation of innate immune responses with an induction of relevant mRNA signals and robust B cell line activation [27]. In addition, Ding and coworkers also proposed a strategy for the construction of a terminal-closed linear gene with a TNA loop modified primer pair through polymerase chain reaction (PCR). The developed linear gene exhibited enhanced enzymatic resistance and serum stability and elicited a potent and persistent EGFP gene expression in eukaryotic cells [28]. Most recently, Yu and coworkers reported TNA-based enzymes with RNA-ligation or RNA-cleavage activities [29,30]. Chaput and coworkers also demonstrated that the introduction of TNA and 2′-fluoroarabino nucleic acid (FANA) modifications into a classic DNAzyme 10–23 (X10-23) significantly enhanced the biological stability and catalytic activity. The obtained X10-23 could be employed to knock down allele-specific mRNA sequences in disease cells and detect the viral pathogen responsible for COVID-19 [[31], [32], [33]]. These findings make TNAs attractive as nucleic acid therapeutics for disease therapy. Nevertheless, the pharmacokinetics and biosafety of TNAs in living organisms are never investigated. Although ASOs reach the target cells/tumors, insufficient tissue penetration and distribution of ASOs result in poor therapeutic efficacy. Therefore, the study of the time course of drug absorption, biodistribution, metabolism, and excretion is of significantly importance.

Herein, we synthesized the fluorophore-labeled TNA oligonucleotides via the well-established cyanoethylphosphoramidite chemistry. The conventional two-dimensional (2D) culture system was used to examine and compare the uptake efficiency of TNAs in a number of cancer cell lines without transfection. Three-dimensional (3D) multicellular tumor spheroids were built to mimic the *in vivo* tumor microenvironment for the study of TNA penetration, distribution, and uptake. Furthermore, animal models are also used to evaluate the *in vivo* pharmacokinetics, biodistribution, and biosafety of TNAs. These studies not only provide a better understanding of the biocompatibility of TNAs with living organisms, but also give an insight into the development of TNA-based delivery systems with improved therapeutic performance as new molecular tools in biomedical applications.

## 2 Materials and methods

### 2.1 Synthesis and characterization of TNA and DNA

Solid-phase synthesis of TNA and DNA oligonucleotide strands was conducted on a BioAutomation MerMade MM6 DNA synthesizer. The TNA was synthesized via previously reported method (Fig. S1) [25]. The TNA and DNA strands were purified through a gel extraction approach and desalted with a Sephadex G-25 column. The successful synthesis of the TNA and DNA strands was confirmed via the Matrix-Assisted Laser Desorption/Ionization Time-of-Flight Mass Spectrometry (MALDI-TOF) analysis and the denaturing polyacrylamide gel electrophoresis (PAGE) analysis. The sequence and mass information of all strands were provided in Table S1.

### 2.2 Fetal Bovine Serum assay

TNA-Cy3 or DNA-Cy3 oligonucleotide strands (1 ​μg) were dissolved in PBS buffer with 10% Fetal Bovine Serum (FBS) to obtain a final concentration of 0.05 ​μg/μL. After that, the samples were incubated at 37 ​°C for various time intervals (0, 1, 2, 4, 8, and 24 ​h). The samples were incubated at 60 ​°C for 15 ​min to denaturalize the enzymes and subsequently characterized with 12% denaturing PAGE analysis in a Maxi Vertical electrophoresis apparatus. Then, the denaturing gel was scanned on a Fujifilm FLA-9000 scanner to detect the Cy3 fluorescence intensity. Afterward, the denaturing gel was stained with StainAll and scanned. The analysis of Cy3 fluorescence intensity and band intensity of TNA or DNA strands was performed with the ImageJ software.

### 2.3 Cell culture

HEK 293 ​cells, HeLa cancer cells, MCF-7 cancer cells, MDA-MB-231 cancer cells, MDA-MB-468 cancer cells, and U87 cancer cells were obtained from the American Type Culture Collection (ATCC, USA). The cells were cultured in DMEM medium with 10% FBS, penicillin (100 U/mL), and streptomycin (100 ​μg/mL) and incubated at 37 ​°C in a humidified 5% CO_2_ atmosphere. MCF-7 ​cells will be selected for a description of the following cell experiments unless indicated.

### 2.4 Cytotoxicity assay

The *in vitro* cytotoxicity of TNA was measured via a standard MTT assay. In general, MCF-7 ​cells were seeded in a 96-well plate at a density of 5000 ​cells/well. After overnight incubation, the culture medium was replaced with fresh medium containing TNA oligonucleotides of various concentrations (0, 0.5, 1, 3, and 5 ​μM). The cells were further incubated for 48 ​h. 20 ​μL MTT (5 ​mg/mL in PBS buffer) was then added to each well and the cells were subsequently incubated for another 4 ​h. After that, the culture medium was discarded followed by 200 ​μL DMSO addition to each well. The plate was incubated in darkness for 30 ​min at 37 ​°C and the absorbance at 590 ​nm was then recorded with a Cytation 3 microplate reader (Bio-Tek). Data were shown as mean ​± ​SD (n ​= ​6).

### 2.5 Confocal fluorescence imaging

MCF-7 ​cells were seeded in 35 ​mm glass-bottomed cell culture dishes at a density of 150,000 ​cells/dish. After overnight incubation, the culture medium was replaced with fresh medium containing TNA-Cy3 oligonucleotides at 0.2 ​μM. The cells were further incubated for various time intervals (0, 2, 4, 8, 12, and 24 ​h). The cells without any treatment were set as 0 ​h. The cells were then stained with Hoechst 33258 ​at r.t. for 20 ​min. After that, the cells were washed with PBS buffer for three times and fixed and analyzed with a Leica TCS SP5 laser confocal scanning microscope. The excitation wavelength for the Cy3 dye is 514 ​nm, and the emission was collected at 550–600 ​nm.

### 2.6 Flow cytometry analysis

MCF-7 ​cells were seeded in 6-well plates at a density of 150,000 ​cells/well. After overnight incubation, the culture medium was replaced with fresh medium containing TNA-Cy3 oligonucleotides at 0.2 ​μM. The cells were further incubated for various time intervals (0, 2, 4, 8, 12, and 24 ​h). The cells without any treatment were set as 0 ​h. After that, the cells were trypsinized and subsequently analyzed on a BD FACS CantoTM II flow cytometer. A PE filter set was employed for the quantitative measurement of the Cy3 signal. Data was analyzed with the FlowJo software.

### 2.7 Three-dimensional (3D) multicellular spheroid preparation

To begin with, 48-well plates coated with agarose were prepared with 1% agarose dissolved in PBS buffer, heated in a microwave oven, and dispensed into each well (0.3 mL/well). After agarose solidification, the plates were exposed to UV light for 2 ​h. U87 ​cells were seeded in an agarose-coated 48-well plate at a cell density of 5000 ​cells/well. The plate was then placed on a shaker at 50 ​rpm for 24 ​h. After that, the cells were incubated in cell incubator with medium renewal after 2 days until the diameter of multicellular spheroids is over 500 ​μm. To obtain multicellular spheroids with different diameters, U87 ​cells were seeded in agarose-coated 48-well plates at various cell densities. MCF-7 multicellular spheroids were also prepared to compare the morphology. During the process, careful operation was taken to avoid the disintegration of multicellular spheroids.

### 2.8 TNA penetration in 3D multicellular spheroids

To study the time-dependent penetration of TNA, the multicellular spheroids were prepared and incubated in culture medium containing TNA-Cy3 oligonucleotides at 0.2 ​μM for various time intervals (0, 8, 24, 48, and 72 ​h). To study the concentration-dependent penetration of TNA, the multicellular spheroids were incubated in culture medium containing TNA-Cy3 oligonucleotides at various concentration (0, 0.1, 0.2, 0.5, 1, and 3 ​μM) for 48 ​h. After that, the multicellular spheroids were analyzed via confocal laser scanning microscopy (CLSM) and Flow Cytometry analysis. For CLSM studies, the multicellular spheroids were imaged every 5 ​μm to determine the penetration depth. The 3D images were also obtained from the reconstruction of all confocal images. For flow cytometry studies, the multicellular spheroids were treated with trypsin for 1 ​h into single cells and these cells were subsequently analyzed on a BD FACS CantoTM II flow cytometer. Data were analyzed with the FlowJo software. To study the relationship of TNA penetration depth with multicellular spheroid diameter, total 60 multicellular spheroids were analyzed regardless of the TNA incubation time and concentration.

### 2.9 Animal studies

The Balb/c mice (6–8 weeks) were purchased from the Laboratory Animal Research Unit (LARU) of City University of Hong Kong and housed in a pathogen-free environment. All animal experiments were approved by the Animal Ethics Committee of City University of Hong Kong and carried out in accordance with the Guidelines for Care and Use of Laboratory Animals of City University of Hong Kong.

### 2.10 Pharmacokinetic studies

To investigate the pharmacokinetics of TNA, purified TNA-Cy5 oligonucleotides were dissolved in PBS buffer and then intravenously injected into Balb/c mice via tail vein at a dose of 4 ​mg/kg. The volume of solution is approximately 150 ​μL depending on the mice weight. Blood samples were harvested from the orbital venous plexus at designated time points (0, 0.5, 2, 8, and 24 ​h) and then centrifuged to get the serum. Mice were anesthetized using the isoflurane anesthesia system all the time during the experiments. After that, the Cy5 fluorescence intensity was determined with a Fluormax-4 Spectrofluorometer. The data were analyzed and shown as the Mean ​± ​SD (n ​= ​3).

### 2.11 Tissue biodistribution

To investigate the *in vivo* tissue biodistribution of TNA, TNA-Cy5 in PBS buffer were intravenously injected into Balb/c mice via tail vein at a dose of 4 ​mg/kg. Afterward, the mice were humanely euthanized, and the selected organs (including heart, lung, liver, spleen, kidneys, brain, intestine, and muscle) were harvested at designated time points (0, 2, 8, 24, and 72 ​h) for *ex vivo* fluorescence imaging. Then, the organs were fixed in 10% formalin and subsequently imaged with a Fujifilm LAS-4000 Luminescent image analyzer. The obtained images were analyzed with the ImageJ software.

### 2.12 In vivo safety analysis

After the intravenous injection of TNA into Balb/c mice via tail vein at a dose of 8 ​mg/kg, blood samples were harvested from the orbital venous plexus at designated time points (0, 0.5, 24, and 72 ​h) were harvested and subsequent hematological analysis was performed using an HLIFE blood analyzer. The obtained mouse blood routine indexes were shown as mean ​± ​SD (n ​= ​3). Blood samples at designated time points (0, 2, 24, and 168 ​h) were also harvested for blood biochemical analysis. Blood biochemical markers including blood urea nitrogen (BUN), creatinine (CREA), and low-density lipoprotein cholesterol (LDL-C) were analyzed using Chemray 800 Automated Chemistry Analyzer and shown as mean ​± ​SD (n ​= ​3). Kidneys and intestines were also harvested at designated time points (0, 2, 24, and 168 ​h) for hematoxylin and eosin (H&E) staining. The H&E staining images were obtained with a Carl Zeiss bright-field Microscope.

### 2.13 Statistics analysis

All data are presented as the mean ​± ​SD. The statistical analysis was performed using the Student's t-test where applicable.

## 3 Results

### 3.1 Physiological stability of TNAs

Fluorescence labelling is a commonly used technique to investigate the structure and function of nucleic acids in cellular environments for molecular biology, bioimaging, biosensing, diagnostics, and therapeutics [[34], [35], [36]]. Generally, cyanine dyes and fluorescein molecules are commonly used for nucleic acid conjugation [[37], [38], [39], [40]]. On the other side, Lacroix et al. recently showed that fluorophores themselves can be dissociated, pass through the cellular membrane, and further localize in intracellular organelles after the fluorophore-labeled DNA/RNA-based structures were degraded by extracellular nucleases [41]. To confirm the structural integrity of the fluorophore-labeled TNAs, the Cy3/Cy5-labeled TNA oligonucleotides were successfully synthesized via standard phosphoramidite chemistry and further characterized by MALDI-TOF (Table S1) and denaturing PAGE analyses (Fig. S2). The sequence we used in this work was just random. There is no specific function. In our previously reported work, it is indicated that TNA strands with other sequences were also able be taken up by different types of living cells [25,26]. In addition, antisense TNA strands without fluorophore labelling can induce targeted gene suppression in cells. These findings suggest that the TNA cellular uptake might not be sequence-, length-, or fluorophore-dependent. Thus, we decide to choose the 15-mer and 17-mer TNAs with random sequences for testing in this work. The length of these selected TNAs was also close to most of the antisense reagents used in other studies. In general, the mobility of the oligonucleotides and other polyelectrolytes on denaturing PAGE analysis is highly depended on the molecular mass and the conformation of oligonucleotides [42]. In the past few years, the conformation of the TNA strand has been evaluated by several research groups [[43], [44], [45], [46]]. Compared with DNA or RNA, TNA is a rigid molecule with compact structure. Therefore, it makes sense that the mobility of TNA strands was slower than that of DNA or RNA strands even if the mass is a bit smaller. This phenomenon is even more obvious in native PAGE analysis [25]. For enzymatic digestion testing, TNA and DNA samples were incubated in 10% FBS in 1 ​× ​PBS buffer for up to 24 ​h at 37 ​°C. As shown in Fig. 1b, the PAGE analysis indicated the rapid degradation of DNA-Cy3 strands in the FBS test. Nevertheless, the TNA strands showed consistent Cy3 fluorescence signal together with strong enzymatic resistance towards FBS incubation. As quantified in Fig. 1c**,** DNA-Cy3 strands degraded completely after 8 ​h incubation of FBS with a half-life of 2.22 ​h. On the contrary, TNA-Cy3 strands did not exhibit significant degradation even after 24 ​h incubation of FBS, which is consistent with previous reports [21,26]. Our results indicated that no enzymatic dissociation of Cy fluorophores from TNAs is observed and the intrinsic structure of TNAs is completely resistant to enzymatic degradation. This finding strongly confirmed the reliability of Cy-labeled TNA oligonucleotides for further tracing and trafficking in cellular and animal studies.

### 3.2 Cellular uptake of TNAs

**Fig. 2:**
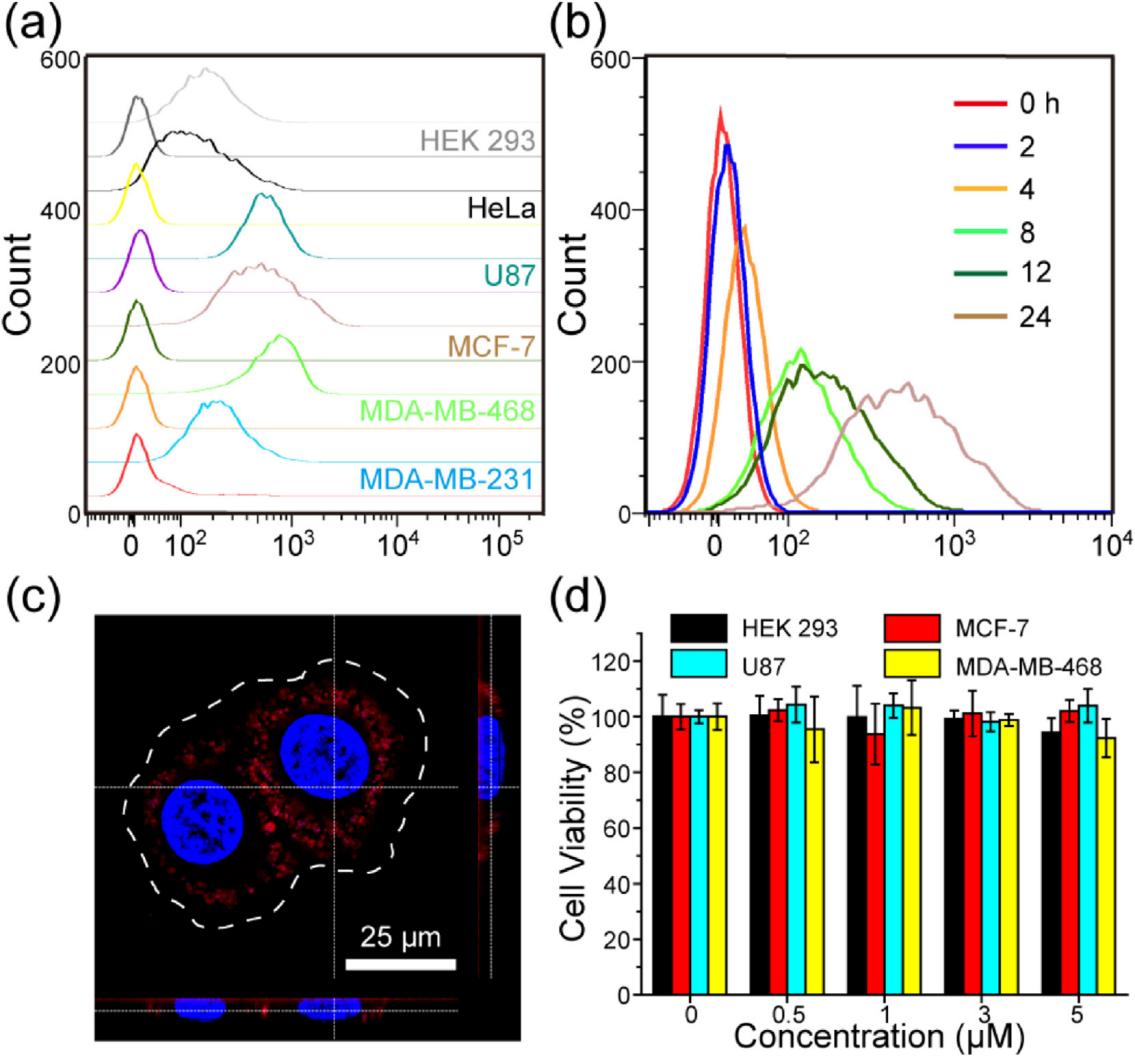
Cellular uptake and cytotoxicity of TNA. (a) Flow Cytometry analysis of the TNA cellular uptake behaviors in various cell lines after 24 ​h incubation. The leftmost peaks refer to the corresponding control cells without TNA incubation. (b) Flow Cytometry analysis of the time-dependent TNA cellular uptake in MCF-7 ​cells (0, 2, 4, 8, 12, and 24 ​h). (c) 3D reconstruction image of MCF-7 ​cells incubated with TNA-Cy3 oligonucleotides (0.2 ​μM) for 12 ​h. The nucleus was stained with Hoechst 33258 dye (blue). The white dotted line represents the cell contour. Scale bar ​= ​25 ​μm. (d) Cytotoxicity assessment of TNA oligonucleotides in HEK 293, MCF-7, U87, and MDA-MB-468 ​cells via MTT assays. Data are shown as the mean ​± ​SD (n ​= ​6).

It was reported that TNA oligonucleotides could penetrate and accumulate inside the cells despite their negative charge [25]. Previous reports also have shown enhanced cellular uptake for other forms of XNAs [37,47,48]. In this work, we compared the TNA uptake kinetics in several types of living cells. Firstly, TNA-Cy3 oligonucleotides were incubated with a number of cell lines for 24 ​h. Their cellular uptake efficiency was characterized and compared using CLSM (Fig. 2a) and flow cytometry (Fig. S3). Our results indicated that different cell lines exhibited different TNA uptake behaviors regarding the uptake amount and distribution pattern. Interestingly, we found that U87, MCF-7, and MDA-MB-468 ​cells show substantially higher cellular uptake efficiency of TNAs when compared with HEK 293, HeLa, and MDA-MB-231 ​cells (Fig. 2a). In particular, the uptake kinetic of TNAs in MCF-7 ​cells was also imaged via CLSM (Fig. S4). No fluorescence signals were observed for the first 2 ​h. Fluorescence signals were first detected at 4 ​h but the signals were mostly found on the outer surface of the cells. Fluorescence signals were clearly observed inside the cells after 8 ​h of incubation and the fluorescence signals further increased with incubation time. Furthermore, a clear shift of cell population was found in flow cytometry analysis at every time point after incubating of TNAs in MCF-7 ​cells (Fig. 2b), revealing the time-dependent cellular uptake of TNA oligonucleotides. We also performed 3D reconstruction based on CLSM images at different laser scanning depths to further confirm the clear stereoscopic image of the localization of TNAs inside the cells, rather than adhesion to the plasma membrane. As indicated in Fig. 2c, the 3D reconstructed image illustrated that the TNA oligonucleotides were inside the cells instead of attaching to the cell surface. In addition, the fluorescence signals were not colocalized within the nucleus, suggesting that the TNA oligonucleotides were distributed in the cytoplasm. We conducted additional experiments to investigate and compare the internalization efficiency and endosomal escape of TNAs after incubation and transfection. To do so, the MDA-MB-231 ​cells were incubated/transfected with TNA-Cy3 for 8 ​h and were then stained with LysoTracker Red before imaging. We did not see a big difference in their internalization intensity in two different treatments as shown in Fig. S5. In addition, about 80% of the Cy3 fluorescence signals were colocalized with the fluorescence signal of LysoTracker Red. On the other hand, there are still some of TNAs’ signal not completely overlapping with the LysoTracker Red. In contrast, the transfected TNAs were not trapped in endosomes inside the cells (Fig. S5), indicating the effective TNA endosomal escape after transfection.

Biocompatibility is another important property of novel materials for biological or biomedical applications. To determine the potential cytotoxicity of TNA oligonucleotides, MTT assay was employed. As shown in Fig. 2d, negligible cytotoxicity was observed in four cell lines used in our study, including HEK 293, MCF-7, U87, and MDA-MB-468 ​cells. The relative cell viability remained to be above 95% even after incubated at a very high TNA concentration for up to 24 ​h. Instead, the incubation of cells with a toxic chemotherapeutic drug doxorubicin (Dox) led to severe cell death (Fig. S6). The substantial cellular uptake and low cytotoxicity of the synthesized TNA oligonucleotides confirmed their excellent biocompatibility, motivating us for further *in vitro* and *in vivo* studies.

### 3.3 TNA penetration in 3D cell culture

**Fig. 3:**
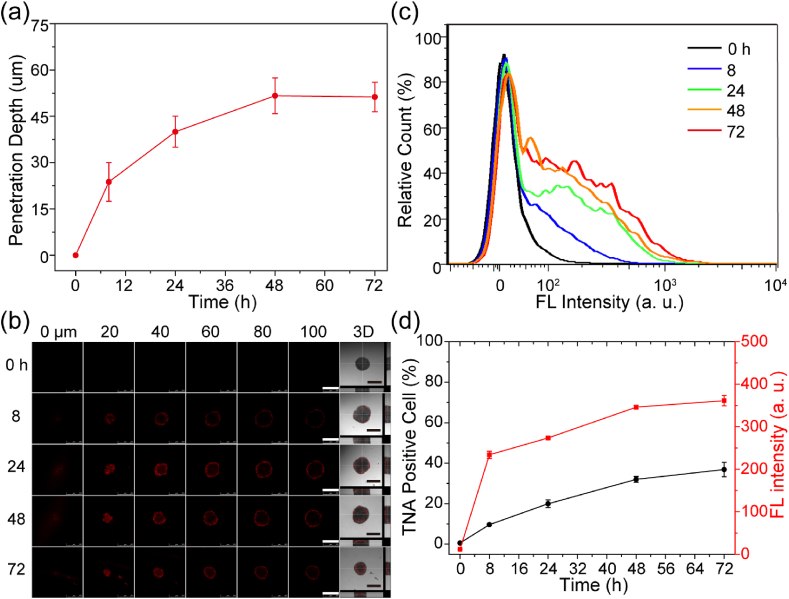
Incubation time-dependent penetration of TNAs in 3D multicellular spheroids. (a) CLSM analysis of penetration depth of the TNA-Cy3 oligonucleotides (0.2 ​μM) in multicellular spheroids against the incubation time (0, 8, 24, 48, and 72 ​h) (n ​= ​4). (b) CLSM images of U87 multicellular spheroids incubated with TNA-Cy3 oligonucleotides (0.2 ​μM) for various time points (0, 8, 24, 48, and 72 ​h). Scale bar ​= ​500 ​μm. (c) Flow cytometry analysis of multicellular spheroids incubated with TNA-Cy3 oligonucleotides (0.2 ​μM) for various time points (0, 8, 24, 48, and 72 ​h). (d) Analysis of the TNA positive cells and Cy3 fluorescence intensity in Fig. 3c.

In general, 2D cultured cells are commonly used for the study of cellular uptake. Unfortunately, the 2D cultured cells are stretched and grown as single layers on a planar solid surface, resulting in a lack of a complex microenvironment, nutritional gradient, intrinsic tensional force, and altered gene expression profiles found in tumor tissues [49]. To address these limitations, we constructed a 3D culture system based on multicellular tumor spheroid model, which offers the unique property to culture cells in a spatially controlled manner, resulting in cell-cell and cell-matrix interactions that closely mimic the physiological environment of native tumors. To begin with, MCF-7 and U87 ​cells were chosen for the establishment of multicellular tumor spheroids according to the previous report [50]. Compared with MCF-7 ​cells, the U87 3D multicellular spheroids exhibited more uniform morphology which would be useful for characterizing the penetration efficiency and hence were chosen for further studies (Fig. S7). The U87 multicellular spheroids with a diameter of ∼300–500 ​μm were then incubated with TNA-Cy3 oligonucleotides and subsequently analyzed with CLSM and flow cytometry. For CLSM studies, the multicellular spheroid was scanned and imaged every 5 ​μm beginning from the very top layer (Fig. S8). For flow cytometry studies, the 3D multicellular spheroids after TNA incubation were trypsinized into single cells and subsequently analyzed. An example of the determination of TNA penetration depth was shown (Fig. S9a). In addition, the 3D image was reconstructed from all the confocal images to present the top, side, and front views of the multicellular spheroid (Fig. S9b). It clearly indicated that the TNAs were able to be taken up by 3D cells without transfection, which is significantly important to antisense cancer therapy studies. As indicated, the TNA penetration depth in the multicellular spheroids increased with incubation time and reached a maximum at 48 ​h (Fig. 3a and b, Fig. S10a). After that, the elongation of incubation time led to minimal increment of TNA penetration. In addition, the flow cytometry analysis revealed similar results regarding the TNA positive cells and Cy3 fluorescence intensity in 3D multicellular spheroids (Fig. 3c and d).

**Fig. 4:**
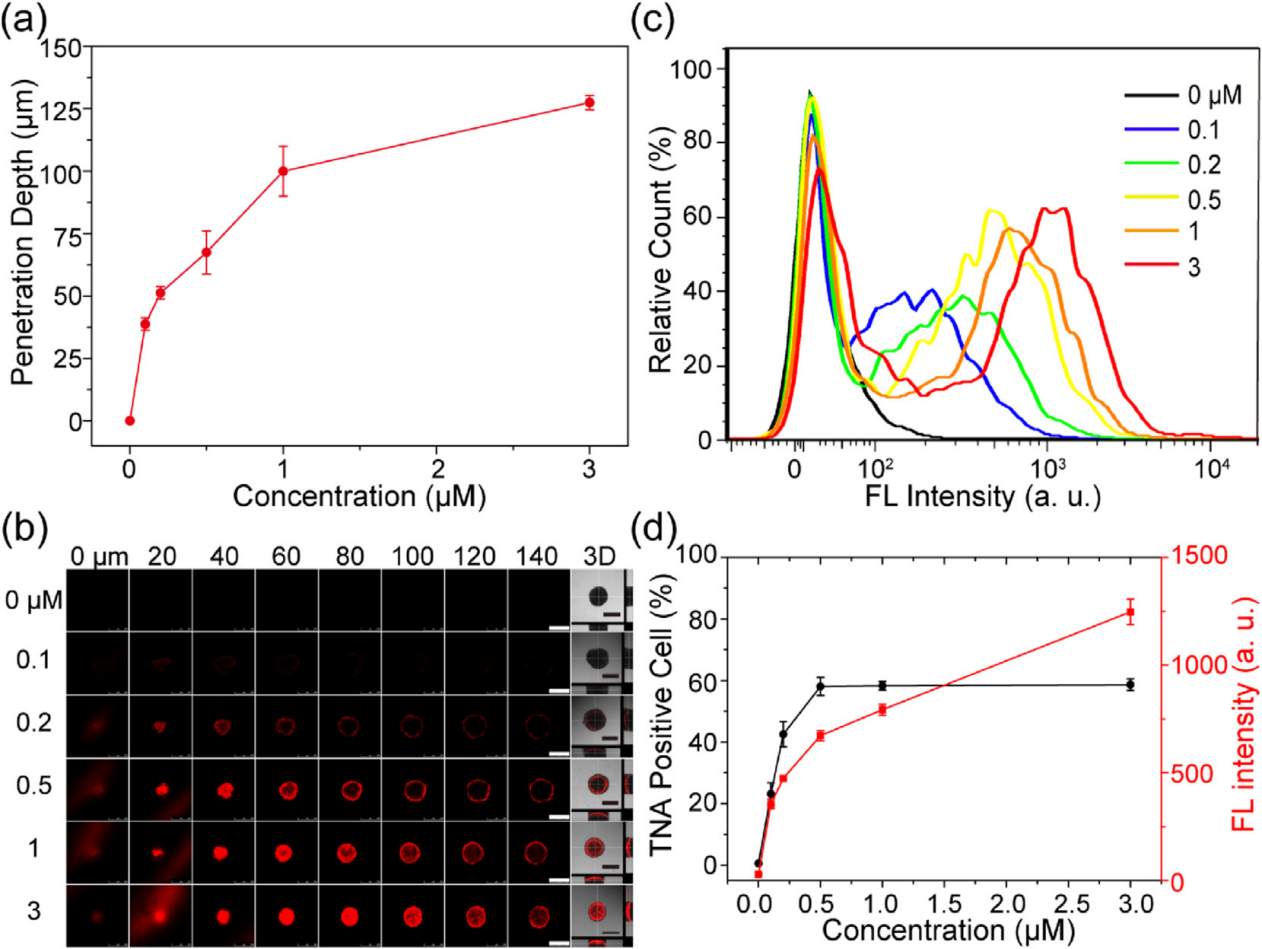
Concentration-dependent penetration of TNAs in 3D multicellular spheroids. (a) CLSM analysis of penetration depth of the TNA in multicellular spheroids against the TNA concentration for 48 ​h (0.1, 0.2, 0.5, 1, and 3 ​μM) (n ​= ​4). (b) CLSM images of U87 multicellular spheroids incubated with TNA-Cy3 oligonucleotides of various concentrations (0, 0.1, 0.2, 0.5, 1, and 3 ​μM) for 48 ​h. Scale bar ​= ​500 ​μm. (c) Flow cytometry analysis of multicellular spheroids incubated with TNA-Cy3 oligonucleotides of various concentrations (0, 0.1, 0.2, 0.5, 1, and 3 ​μM) for 48 ​h. (d) Analysis of the TNA positive cells and Cy3 fluorescence intensity in Fig. 4c.

On the other hand, the results showed that TNA penetration depth also increased with TNA concentration with 48 ​h incubation (Fig. 4a and b, Fig. S10b). Furthermore, as observed, we expect that the maximum penetration depth would be reached at concentration higher than 3 ​μM. In addition, the flow cytometry results indicated the continuous enhancement in Cy3 fluorescence intensity by increasing the TNA concentration (Fig. 4c and d). These results indicated the time-, and concentration-dependent TNA penetration in multicellular spheroids, which are consistent with previous reports studying the penetration of oligonucleotides and nanoparticles in the multicellular spheroids [51,52].

**Fig. 5:**
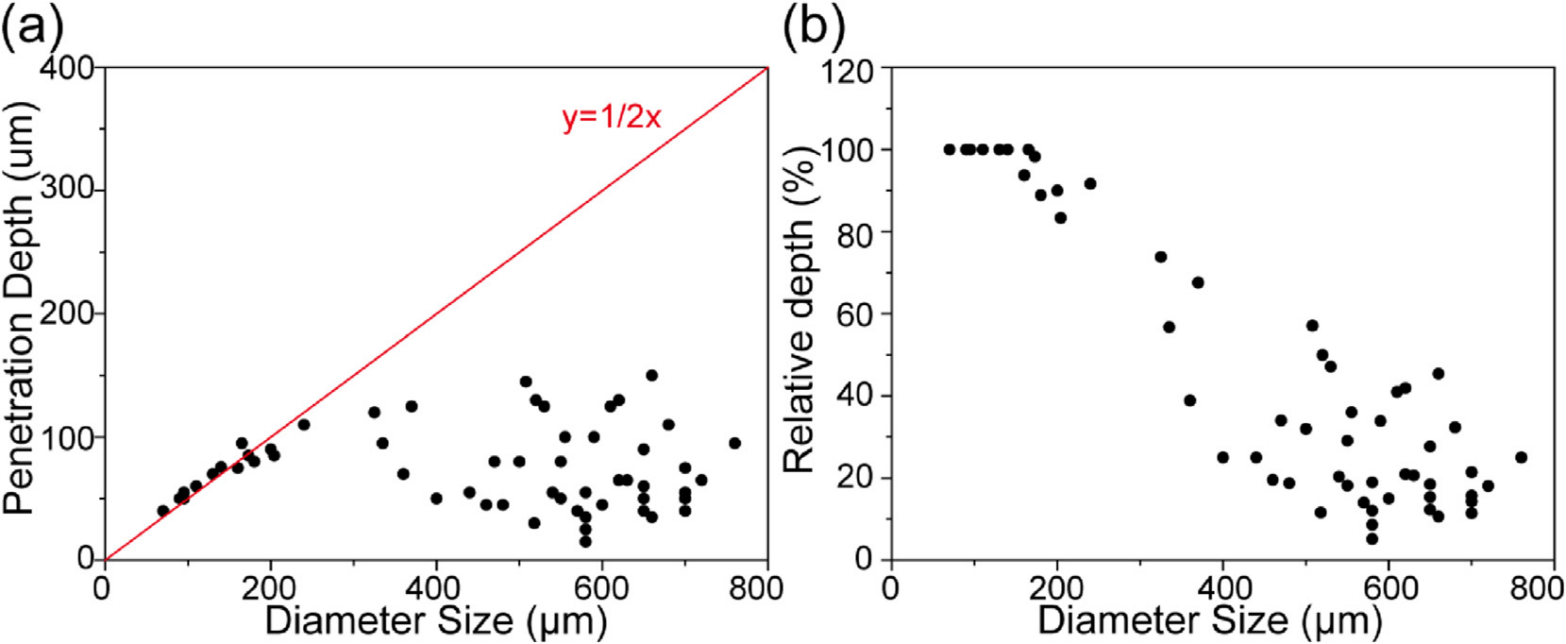
Spheroid size-dependent penetration of TNAs in 3D multicellular spheroids. (a) Plotted coordinates showing the diameter size and corresponding TNA penetration depth of selected 60 multicellular spheroids. The red line indicates the radius. (b) The relative depth of TNA penetration in the multicellular spheroids calculated from Fig. 5a. The relative depth is defined as the ratio of the TNA penetration depth to the radius of the multicellular spheroid.

We also investigated the relationship between the TNA penetration depth and the spheroid diameter. A total of 60 multicellular spheroids were analyzed regardless of the TNA concentration and incubation time. As shown, the TNAs could penetrate efficiently throughout the whole multicellular spheroid (100% relative depth) when the size of the spheroid is relatively small (below 200 ​μm) (Fig. 5a and b, Fig. S11). Nevertheless, when the spheroid diameter increased, the TNA penetration is limited to a certain extent as raising the TNA concentration and incubation time no longer led to the TNA diffusion throughout the multicellular spheroid (<100% relative depth). We found that the maximum penetration depth was about 150–170 ​μm for TNA oligonucleotides (Fig. 3a).

### 3.4 *In vivo* pharmacokinetics and tissue biodistribution

**Fig. 6:**
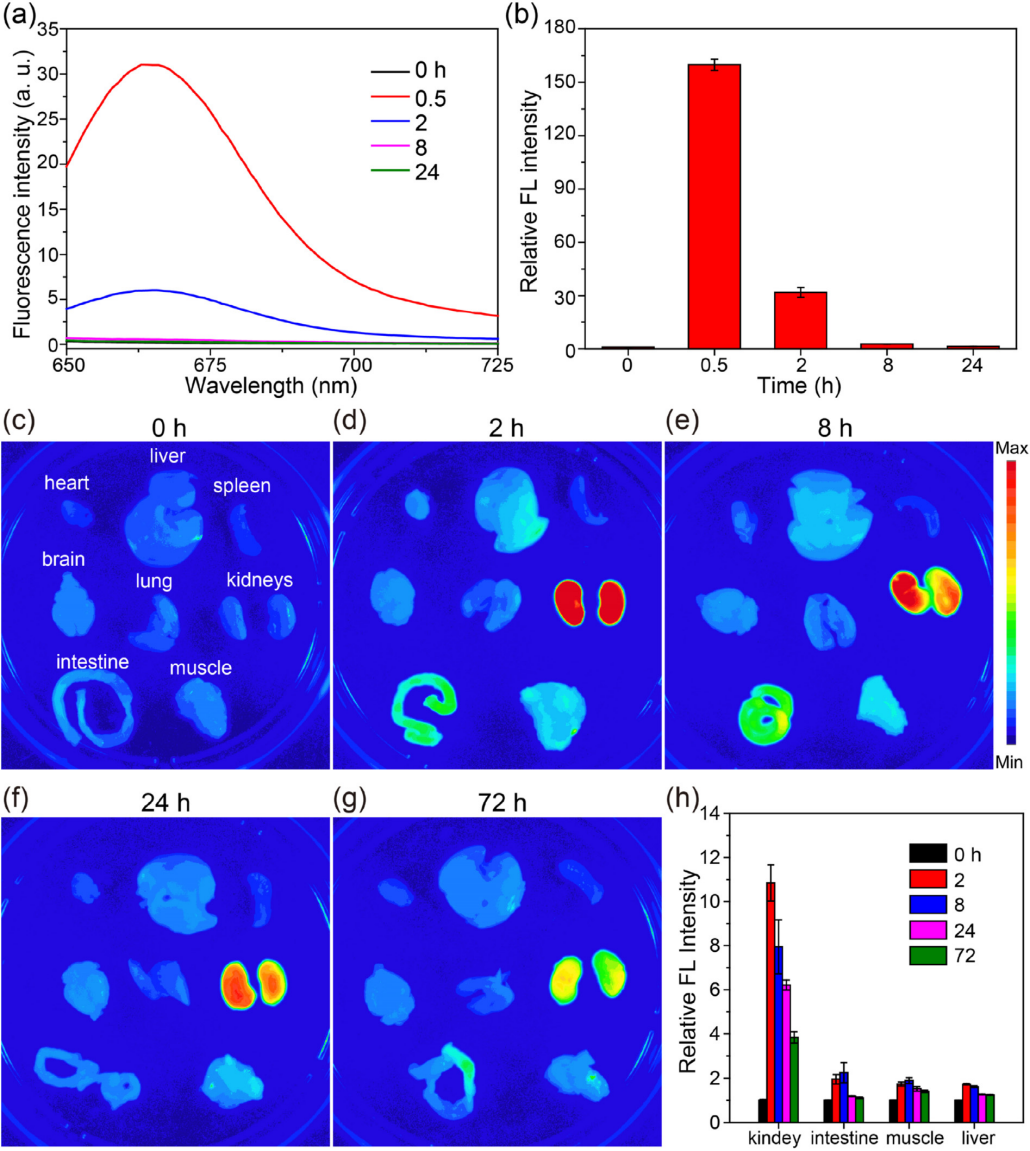
(a) The Cy5 fluorescence spectra of blood serum samples harvested at designated time points (0, 0.5, 2, 8, and 24 ​h) after intravenous injection of TNA-Cy5 oligonucleotides. (b) The relative fluorescence intensity of blood serum samples obtained from Fig. 6a. (c–g) Fluorescence imaging of organs harvested at different time intervals (0, 2, 8, 24, and 72 ​h) after the intravenous injection of TNA-Cy5 oligonucleotides. (h) The relative fluorescence intensity in different organs at different time points (0, 2, 8, 24, and 72 ​h) after intravenous injection of TNA-Cy5 oligonucleotides.

Motivated by the efficient penetration and uptake of TNA oligonucleotides in *in vitro* tumor models, we further investigated their interactions with animals. Firstly, body clearance experiments were conducted to examine the *in vivo* pharmacokinetics and biodistribution of TNAs. TNA oligonucleotides were first labeled with Cy5 (TNA-Cy5) and were then injected intravenously into Balb/c mice. The blood samples were collected at different time points, and the Cy5 fluorescence signals were then measured. As shown in Fig. 6a and b, the fluorescence intensity of Cy5 in the blood increased drastically (by over 150-fold) within 0.5 ​h after systemic administration of TNA-Cy5 oligonucleotides. Afterward, the Cy5 signal displayed a rapid decline. More than 97% of TNAs vanished at 8 ​h. The results indicated the rapid clearance of TNAs during blood circulation.

We argued that the fast removal of the TNA oligonucleotides was due to the rapid transfer from the blood into tissues, as other types of oligonucleotides do [53]. Thus, the major organs including the lung, heart, brain, liver, spleen, kidneys, intestine, and muscle were harvested and visualized at different time by *ex vivo* fluorescence imaging. As shown, the kidneys showed the strongest fluorescence at 2 ​h after the administration of TNAs, suggesting the rapid and dominant uptake and accumulation of TNAs in the kidneys (Fig. 6c–g). Distribution of TNAs in the organs of the intestine, muscle, and liver was also observed but with much lower level (Fig. 6h). Furthermore, TNAs in the kidneys decreased by 65% at 72 ​h compared with that at 2 ​h (Fig. 6h), suggesting the excretion of TNAs from the body through the renal filtration system. The results are in good agreement with the preferential ultrafiltration of other types of oligonucleotides by the kidney [[54], [55], [56]]. More importantly, the primary distribution of TNAs in kidneys is consistent with the previous work reported by Yu and coworkers [24].

### 3.5 *In vivo* biosafety of TNAs

**Fig. 7:**
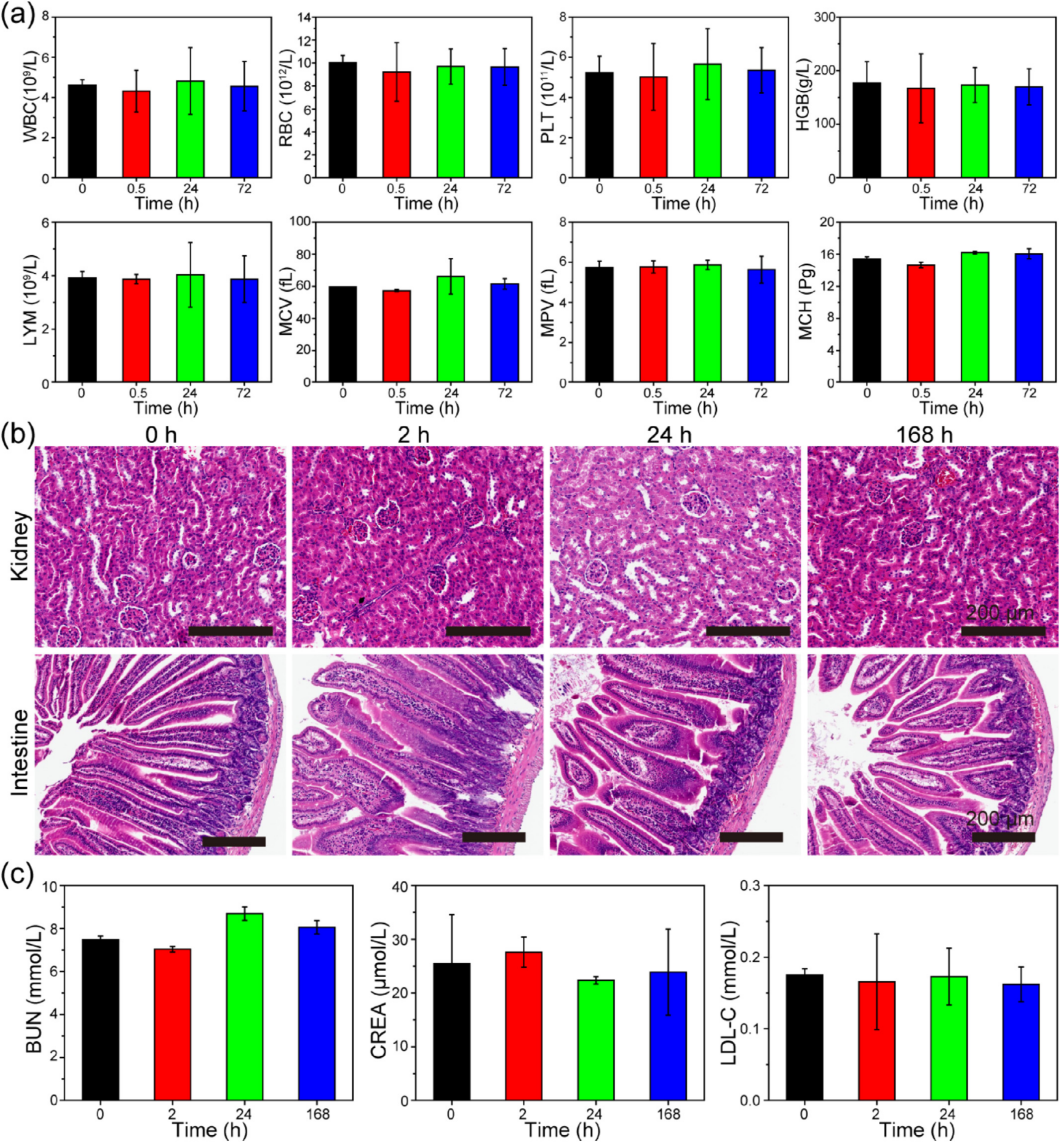
*In vivo* biosafety of TNA oligonucleotides. (a) The blood routine indexes of mice at 0 ​h, 0.5 ​h, 24 ​h, and 72 ​h after TNAs intravenous injection (WBC: white blood cells; RBC: red blood cells; PLT: platelet; HGB: hemoglobin (HGB); LYM: lymphocytes; MCV: mean corpuscular volume; MPV: mean platelet volume; MCH: mean corpuscular hemoglobin). Error bars indicate standard deviation (n ​= ​3). (b) Histology H&E staining of kidney and intestine harvested at 0 ​h, 2 ​h, 24 ​h, and 168 ​h after TNAs intravenous injection. Scale bar ​= ​200 ​μm. (c) Blood biochemistry analysis of renal function at 0 ​h, 2 ​h, 24 ​h, and 168 ​h after TNAs intravenous injection. (BUN: blood urea nitrogen; CREA: creatinine; LDL-C: low-density lipoprotein cholesterol). Error bars indicate standard deviation (n ​= ​3).

So far, TNA oligonucleotides exhibited attractive characteristics such as remarkable physiological stability, high binding affinity towards complementary RNAs, and enhanced cellular uptake, making TNAs promising candidates for the development of diagnostic and therapeutic agents. Nevertheless, the concern of the long-term toxicity of developed XNAs still limits their biological and biomedical applications [9,14]. To examine the *in vivo* biosafety of the TNA oligonucleotides, blood samples were collected at 2, 24, and 168 ​h after intravenous injection of TNAs. As shown in Fig. 7a, the blood routine indexes including the white blood cells (WBC), red blood cells (RBC), platelet (PLT), hemoglobin (HGB), lymphocytes (LYM), mean corpuscular volume (MCV), mean platelet volume (MPV), and mean corpuscular hemoglobin (MCH) indicated negligible differences between sample groups and control groups (untreated mice (0 ​h)), suggesting the TNA administration will not lead to obvious pathological changes. Since the TNA oligonucleotides preferentially accumulated in the kidneys and some in the intestine, we conducted experiments to investigate the potential side effects of TNAs to these organs, particularly to the kidney. As shown in Fig. 7b, the histology hematoxylin and eosin (H&E) staining of the kidney and intestine slices for TNA-treated mice revealed no appreciable histopathological abnormality of the tissues with the tested dose. In addition, we performed blood biochemistry analysis and showed that blood urea nitrogen (BUN), creatinine (CREA), and low-density lipoprotein cholesterol (LDL-C) in TNA-treated mice displayed no significant variations compared with control groups, suggesting that the TNA uptake and accumulation in the kidneys induce no acute renal injury and insufficiency (Fig. 7c). Taken together, these data strongly confirmed the excellent biocompatibility of TNA oligonucleotides for practical applications in the biological environment.

## 4 Discussion

TNA possesses attractive properties of chemical simplicity, high binding specificity towards DNA/RNA, tertiary structure folding capability, and excellent biological stability, holding great potential in biology study and biomedical applications [21,22,29]. So far, TNA has been widely used in various biomedical applications such as aptamer selection [22,29], XNAzyme discovery [[29], [30], [31], [32], [33]], gene regulation [25,26,28], and immunotherapy [24,27]. In this work, we systematically investigated the interactions of TNA oligonucleotides with living organisms, including cellular uptake, penetration in 3D cell culture, pharmacokinetics, tissue biodistribution, and *in vivo* biosafety for the first time.

Nucleic acid modification with fluorescence dyes is a mature technique in chemical synthesis and has been widely used for nucleic acid tracking and trafficking in biology. However, Lacroix et al. recently showed that fluorophores themselves can be released, pass through the cellular membrane, and further localize in intracellular organelles after the fluorophore-labeled DNA/RNA-based structures were degraded by extracellular nucleases [41]. To ensure no misleading fluorescent signals to be obtained for the *in vitro* and *in vivo* studies via fluorescence imaging, serum stability of the fluorophore-labeled TNA oligonucleotides was investigated and compared. The FBS assays indicated the strong enzymatic resistance of TNA oligonucleotides compared with rapid degraded DNA. TNA-Cy3 oligonucleotides remained integral even after incubation with FBS for 24 ​h, however, corresponding DNA-Cy3 oligonucleotides displayed complete degradation in 8 ​h incubation (Fig. 1b and c). Because of the strong biological stability of TNA, we can eliminate the concern of degradation-caused misleading false signals in fluorescent nucleic acid imaging.

We used the CLSM and flow cytometry techniques to study the cellular uptake property of TNAs. Different cell lines displayed distinct behaviors in TNA uptake amount and distribution pattern (Fig. 2a). Our findings were in good agreement with the results of phosphorothioate oligonucleotides [37,53]. In general, factors including length and sequence of oligonucleotides, chemical modifications, and the cell physiological state would affect the cellular uptake of oligonucleotide [37,57]. In previously reported work, it is indicated that TNA strands with different lengths and sequences exhibited effective cell penetration [25,26], nevertheless, the number of tested TNAs are still limited. Alternatively, Stein et al. also demonstrated that the naked LNA can be efficiently taken up by living cells via the “gymnosis” process without the use of transfection reagents and enables the sequence-specific gene silencing in different types of cells [58]. Given that this is a carrier-free delivery system, gymnotic silencing would offer more antisense oligonucleotide-specific phenotypes. Additionally, a cellular response is easily to be interpreted as this response is only related to the added ASOs [59]. As the cellular TNA uptake was mainly attributed to endocytosis, the differences in TNA uptake for various cell lines might be due to the differentially expressed receptors and the distinct membrane lipid composition on the cell surface [26,37,54]. Compared with siRNAs or other ASOs, TNAs exhibited effective uptake in living cells without the aid of carriers [14,25]. This suggests that TNA can be potentially used as a simple and cost-effective therapeutic agent for cancer therapy. We also compared the TNA internalization efficiency and endosomal escape in incubation and transfection methods. The observations confirmed that majority of the TNAs were trapped in endosomes inside the cells. In fact, our CLSM results are in a good agreement with the results of its uptake mechanism studies. We have previously demonstrated that cellular uptake of TNA was dominantly attributed to a temperature- and energy-dependent endocytotic pathway [26]. The mechanism of oligonucleotide release from endosomes are still unclear, but the maturation from early to late endosomes is reported to play an important role [59]. In addition, the transfection reagents do not contribute much to the internalization efficiency, but they can remarkably boost the endosomal escape of TNAs and alter their intracellular localization. Therefore, the development of delivery systems for efficient TNAs transfection can significantly improve their therapeutic potency.

Although we confirmed the time-dependent cellular uptake of TNAs into living cells, the experiments were performed on 2D culture systems. It is generally agreed that the penetration and uptake performance of therapeutic agents can be quite different in 2D cultures and 3D tumor tissues [49]. Accordingly, we prepared U87 multicellular spheroids as 3D cell culture model to mimic the tumor physiological environment and studied the TNA penetration property via CLSM and flow cytometry analysis. The results indicated the time-, and concentration-dependent TNA penetration in multicellular spheroids (Figs. 3 and 4). Increasing the incubation time and TNA concentration led to the increment of TNA penetration depth. Nevertheless, the TNA penetration is limited in relatively large spheroids. Raising the TNA incubation time and concentration could not continuously enhance the TNA penetration and eventually led to a saturated TNA penetration (Fig. 5). This observation may attribute to the high interstitial fluid pressure and complicated composition of the extracellular matrix in relatively large multicellular spheroids, which have been reported to hinder the delivery of drugs and biomacromolecules [60,61]. To improve the therapeutic effects, future work can focus on the development of TNA-based agents with enhanced penetration property with targeting ligands [8,51].

In this study, we used TNA-Cy5 oligonucleotides to study the *in vivo* pharmacokinetics and tissue biodistribution. After intravenous administration, TNAs displayed rapid clearance with over 97% of them removed in 8 ​h during blood circulation (Fig. 6a and b). The *ex vivo* fluorescence imaging showed the rapid transfer of the TNA oligonucleotides from the blood into tissues, especially in kidneys (Fig. 6d). We believe that the TNA accumulation in the kidneys was possibly due to the preferential uptake by the kidney proximal tubule cells [40,62]. TNA oligonucleotides for gene regulation have been reported in the past few years [[25], [26], [27], [28],[30], [31], [32]]. For example, Chaput and coworkers demonstrated the TNA CPG strands can activate innate immune responses with induction of relevant mRNAs and B-cell activation [27]. Very recently, Yu et al. reported the *in vitro* isolation of TNA-based enzymes with RNA-cleaving activity [30]. One selected TNA enzyme, Tz1, can specifically cleave the mutant epidermal growth factor receptor (EGFR) RNA substrate with a single point mutation. They also demonstrated the selective inhibition of the mutant EGFR proteins and subsequent suppressed cell viability in H1975 cancer cells. However, these developed TNA-based reagents are mostly utilized in in vitro studies. Considering the huge differences between *in vitro* and *in vivo* conditions, the bioavailability of TNA reagents is still a major challenge for therapeutic applications. Notably, the dominant accumulation and rapid renal clearance of TNAs in the kidneys might lead to insufficient delivery of therapeutic TNAs in the targeted cells/tissues though the enzymatic degradation of TNA is not a concern. Encouragingly, a recent work by Yu and coworkers demonstrated the altered biodistribution of TNA strands with specific protein-binding ability [24]. The data indicated that the selected TNA aptamer N5 could specifically accumulate at the tumor site while the scrambled sequence displayed favoured accumulation in the kidneys in a mouse model. Thus, selections of TNA strands with targeting ability or modifications of TNAs with targeting moieties would be necessary for the efficient delivery and accumulation of therapeutic TNAs in interested cells or organ tissues to achieve improved therapeutic efficiency and reduced side effects [24,51].

We also investigated the *in vivo* biosafety of TNA oligonucleotides. The mice blood routine indexes indicated insignificant differences after intravenous injection (Fig. 7a). No histopathological abnormalities were observed in the H&E staining of kidneys and intestines (Fig. 7b). In addition, the blood biochemistry analysis suggested no acute renal injury and no renal insufficiency caused by preferential TNA uptake and accumulation of TNA in the kidneys (Fig. 7c). Notably, it is of significance investigating the potential recognition of TNAs by the immune system and the potential consequences of these recognition events. This will be another piece of TNA work on-going in our group. To plan for this long-term TNA toxicity studies, we are interested in investigating oligonucleotide accumulation effects, proinflammatory mechanisms, and binding interactions with proteins [63]. For example, the interactions of TNA oligonucleotides with immune system and potential proinflammatory activities needs to be well studied. In addition, treatment of higher doses or multiple exposures of TNA oligonucleotides to animals might enhance the accumulation-related effects and resulted in long-term histopathological abnormalities. Therefore, a better understanding of TNA toxicity is beneficial for the future development of TNA-based reagents in biological and biomedical applications.

## 5 Conclusions

Compared with siRNAs or other ASOs, synthetic TNAs exhibited excellent biological stability, low cytotoxicity, and substantial uptake in living cells without the aid of carriers. Using U87 3D multicellular spheroids as a model system, TNAs showed their ability to penetrate efficiently throughout the whole multicellular spheroid as a function of incubation time and concentration when the size of the spheroid is relatively small. Additionally, TNAs could be safely administrated into Balb/c mice and most of them distributed in the kidneys where they supposed to excrete from the body through the renal filtration system. Accumulation of TNAs in kidneys induced no pathological changes, and no acute structural and functional damage in renal systems. The favourable pharmacokinetics and biosafety of TNA make it attractive as a highly biocompatible and cost-effective nucleic acid-based therapeutic agent for practical biological and biomedical applications. To fully validate the utility of TNAs for disease therapy, investigation of TNA strands functionalized with targeting ligands is necessary.

## Credit author statement

Fei Wang: Investigation; Methodology; Data curation; Formal analysis; Visualization; Writing – original draft, Ling Sum Liu: Investigation; Methodology, Pan Li, Investigation; Methodology, Cia Hin Lau: Investigation; Methodology, Hoi Man Leung: Investigation; Methodology, Y Rebecca Chin: Resources, Chung Tin: Resources, Conceptualization; Funding acquisition, Pik Kwan Lo: Conceptualization; Funding acquisition; Project administration; Supervision; Writing – review & editing.

## Data availability

The data sets used and/or analyzed during the current study are available from the corresponding author on reasonable request.

## Declaration of competing interest

The authors declare that they have no known competing financial interests or personal relationships that could have appeared to influence the work reported in this paper.

## References

[1] Opalinska J.B., Gewirtz A.M. (2002). Nucleic-acid therapeutics: basic principles and recent applications. Nat. Rev. Drug Discov..

[2] Weng Y.H., Huang Q.Q., Li C.H., Yang Y.F., Wang X.X., Yu J., Huang Y.Y., Liang X.J. (2020). Improved nucleic acid therapy with advanced nanoscale biotechnology. Mol. Ther. Nucleic Acids.

[3] Nguyen J., Szoka F.C. (2012). Nucleic acid delivery: the missing pieces of the puzzle?. Accounts Chem. Res..

[4] Setten R.L., Rossi J.J., Han S.P. (2019). The current state and future directions of RNAi-based therapeutics. Nat. Rev. Drug Discov..

[5] Wagner H.J., Weber W., Fussenegger M. (2021). Synthetic biology: emerging concepts to design and advance adeno-associated viral vectors for gene therapy. Adv. Sci..

[6] Kotterman M.A., Chalberg T.W., Schaffer D.V. (2015). Viral vectors for gene therapy: translational and clinical outlook. Annu. Rev. Biomed. Eng..

[7] Chin Y.R., Yoshida T., Marusyk A., Beck A.H., Polyak K., Toker A. (2014). Targeting Akt3 signaling in triple-negative breast cancer. Cancer Res..

[8] Lin G.M., Li L., Panwar N., Wang J., Tjin S.C., Wang X.M., Yong K.T. (2018). Non-viral gene therapy using multifunctional nanoparticles: status, challenges, and opportunities. Coord. Chem. Rev..

[9] Khvorova A., Watts J.K. (2017). The chemical evolution of oligonucleotide therapies of clinical utility. Nat. Biotechnol..

[10] Tam D.Y., Ho J.W.-T., Chan M.S., Lau C.H., Chang T.J.H., Leung H.M., Liu L.S., Wang F., Chan L.L.H., Tin C. (2020). Penetrating the blood–brain barrier by self-assembled 3D DNA nanocages as drug delivery vehicles for brain cancer therapy. ACS Appl. Mater. Interfaces.

[11] Wang F., Chen H., Liu Z., Mi F., Fang X., Liu J., Wang M., Lo P.K., Li Q. (2019). Conjugated polymer dots for biocompatible siRNA delivery. New J. Chem..

[12] McKenzie L.K., El-Khoury R., Thorpe J.D., Damha M.J., Hollenstein M. (2021). Recent progress in non-native nucleic acid modifications. Chem. Soc. Rev..

[13] Wang F., Li P., Chu H.C., Lo P.K. (2022). Nucleic acids and their analogues for biomedical applications. Biosensors.

[14] Morihiro K., Kasahara Y., Obika S. (2017). Biological applications of xeno nucleic acids. Mol. Biosyst..

[15] Stein D., Foster E., Huang S.B., Weller D., Summerton J. (1997). A specificity comparison of four antisense types: morpholino, 2'-O-methyl RNA, DNA, and phosphorothioate DNA. Antisense Nucleic Acid Drug Dev..

[16] Koppelhus U., Awasthi S.K., Zachar V., Holst H.U., Ebbesen P., Nielsen P.E. (2002). Cell-dependent differential cellular uptake of PNA, peptides, and PNA-peptide conjugates. Antisense Nucleic Acid Drug Dev..

[17] Swayze E.E., Siwkowski A.M., Wancewicz E.V., Migawa M.T., Wyrzykiewicz T.K., Hung G., Monia B.P., Bennett C.F. (2007). Antisense oligonucleotides containing locked nucleic acid improve potency but cause significant hepatotoxicity in animals. Nucleic Acids Res..

[18] Chen C.M., Yang Z.J., Tang X.J. (2018). Chemical modifications of nucleic acid drugs and their delivery systems for gene-based therapy. Med. Res. Rev..

[19] Schoning K.U., Scholz P., Guntha S., Wu X., Krishnamurthy R., Eschenmoser A. (2000). Chemical etiology of nucleic acid structure: the alpha-threofuranosyl-(3 '-> 2 ') oligonucleotide system. Science.

[20] Yu H.Y., Zhang S., Chaput J.C. (2012). Darwinian evolution of an alternative genetic system provides support for TNA as an RNA progenitor. Nat. Chem..

[21] Culbertson M.C., Temburnikar K.W., Sau S.P., Liao J.Y., Bala S., Chaput J.C. (2016). Evaluating TNA stability under simulated physiological conditions. Bioorg. Med. Chem. Lett.

[22] Dunn M.R., McCloskey C.M., Buckley P., Rhea K., Chaput J.C. (2020). Generating biologically stable TNA aptamers that function with high affinity and thermal stability. J. Am. Chem. Soc..

[23] Rangel A.E., Chen Z., Ayele T.M., Heemstra J.M. (2018). In vitro selection of an XNA aptamer capable of small-molecule recognition. Nucleic Acids Res..

[24] Li X.T., Li Z., Yu H.Y. (2020). Selection of threose nucleic acid aptamers to block PD-1/PD-L1 interaction for cancer immunotherapy. Chem. Commun..

[25] Liu L.S., Leung H.M., Tam D.Y., Lo T.W., Wong S.W., Lo P.K. (2018). Alpha-L-threose nucleic acids as biocompatible antisense oligonucleotides for suppressing gene expression in living cells. ACS Appl. Mater. Interfaces.

[26] Wang F., Liu L.S., Lau C.H., Han Chang T.J., Tam D.Y., Leung H.M., Tin C., Lo P.K. (2019). Synthetic α-l-Threose nucleic acids targeting BcL-2 show gene silencing and in vivo antitumor activity for cancer therapy. ACS Appl. Mater. Interfaces.

[27] Lange M.J., Burke D.H., Chaput J.C. (2019). Activation of innate immune responses by a CpG oligonucleotide sequence composed entirely of threose nucleic acid. Nucleic Acid Therapeut..

[28] Lu X.H., Wu X.H., Wu T.T., Han L., Liu J.B., Ding B.Q. (2020). Efficient construction of a stable linear gene based on a TNA loop modified primer pair for gene delivery. Chem. Commun..

[29] Wang Y., Wang Y.Y., Song D.F., Sun X., Zhang Z., Li X.T., Li Z., Yu H.Y. (2021). A threose nucleic acid enzyme with RNA ligase activity. J. Am. Chem. Soc..

[30] Wang Y., Wang Y., Song D., Sun X., Li Z., Chen J.-Y., Yu H. (2022). An RNA-cleaving threose nucleic acid enzyme capable of single point mutation discrimination. Nat. Chem..

[31] Wang Y.J., Nguyen K., Spitale R.C., Chaput J.C. (2021). A biologically stable DNAzyme that efficiently silences gene expression in cells. Nat. Chem..

[32] Nguyen K., Wang Y.J., England W.E., Chaput J.C., Spitale R.C. (2021). Allele-specific RNA knockdown with a biologically stable and catalytically efficient XNAzyme. J. Am. Chem. Soc..

[33] Yang K.F., Chaput J.C. (2021). REVEALR: a multicomponent XNAzyme-based nucleic acid detection system for SARS-CoV-2. J. Am. Chem. Soc..

[34] Asseline U. (2006). Development and applications of fluorescent oligonucleotides. Curr. Org. Chem..

[35] Liu L.S., Wang F., Ge Y.H., Lo P.K. (2021). Recent developments in aptasensors for diagnostic applications. ACS Appl. Mater. Interfaces.

[36] Wang X.X., Zhu L.J., Li S.T., Zhang Y.Z., Liu S.Y., Huang K.L., Xu W.T. (2021). Fluorescent functional nucleic acid: principles, properties and applications in bioanalyzing. TrAC Trends Anal. Chem. (Reference Ed.).

[37] Crooke S.T., Wang S.Y., Vickers T.A., Shen W., Liang X.H. (2017). Cellular uptake and trafficking of antisense oligonucleotides. Nat. Biotechnol..

[38] Loke S.L., Stein C.A., Zhang X.H., Mori K., Nakanishi M., Subasinghe C., Cohen J.S., Neckers L.M. (1989). Characterization of oligonucleotide transport into living cells. Proc. Natl. Acad. Sci. U.S.A..

[39] Wang S.Y., Allen N., Vickers T.A., Revenko A.S., Sun H., Liang X.H., Crooke S.T. (2018). Cellular uptake mediated by epidermal growth factor receptor facilitates the intracellular activity of phosphorothioate-modified antisense oligonucleotides. Nucleic Acids Res..

[40] Juliano R.L., Ming X., Nakagawa O. (2012). Cellular uptake and intracellular trafficking of antisense and siRNA oligonucleotides. Bioconjugate Chem..

[41] Lacroix A., Vengut-Climent E., de Rochambeau D., Sleiman H.F. (2019). Uptake and fate of fluorescently labeled DNA nanostructures in cellular environments: a cautionary tale. ACS Cent. Sci..

[42] Viovy J.-L. (2000). Electrophoresis of DNA and other polyelectrolytes: physical mechanisms. Rev. Mod. Phys..

[43] Pallan P.S., Wilds C.J., Wawrzak Z., Krishnamurthy R., Eschenmoser A., Egli M. (2003). Why does TNA cross-pair more strongly with RNA than with DNA? An answer from X-ray analysis. Angew. Chem. Int. Ed..

[44] Ebert M.O., Mang C., Krishnamurthy R., Eschenmoser A., Jaun B. (2008). The structure of a TNA-TNA complex in solution: NMR study of the octamer duplex derived from alpha-(L)-Threofuranosyl-(3 '-2 ')-CGAATTCG. J. Am. Chem. Soc..

[45] Anosova I., Kowal E.A., Sisco N.J., Sau S., Liao J.Y., Bala S., Rozners E., Egli M., Chaput J.C., Van Horn W.D. (2016). Structural insights into conformation differences between DNA/TNA and RNA/TNA chimeric duplexes. Chembiochem.

[46] Muniz M.I., Lackey H.H., Heemstra J.M., Weber G. (2020). DNA/TNA mesoscopic modeling of melting temperatures suggests weaker hydrogen bonding of CG than in DNA/RNA. Chem. Phys. Lett..

[47] Zhou P., Wang M.M., Du L., Fisher G.W., Waggoner A., Ly D.H. (2003). Novel binding and efficient cellular uptake of guanidine-based peptide nucleic acids (GPNA). J. Am. Chem. Soc..

[48] Yamamoto T., Yahara A., Waki R., Yasuhara H., Wada F., Harada-Shiba M., Obika S. (2015). Amido-bridged nucleic acids with small hydrophobic residues enhance hepatic tropism of antisense oligonucleotides in vivo. Org. Biomol. Chem..

[49] Kunz-Schughart L.A. (1999). Multicellular tumor spheroids: intermediates between monolayer culture and in vivo tumor. Cell Biol. Int..

[50] Kelm J.M., Timmins N.E., Brown C.J., Fussenegger M., Nielsen L.K. (2003). Method for generation of homogeneous multicellular tumor spheroids applicable to a wide variety of cell types. Biotechnol. Bioeng..

[51] Carver K., Ming X., Juliano R.L. (2014). Multicellular tumor spheroids as a model for assessing delivery of oligonucleotides in three dimensions. Mol. Ther. Nucleic Acids.

[52] Tchoryk A., Taresco V., Argent R.H., Ashford M., Gellert P.R., Stolnik S., Grabowska A., Garnett M.C. (2019). Penetration and uptake of nanoparticles in 3D tumor spheroids. Bioconjugate Chem..

[53] Geary R.S., Norris D., Yu R., Bennett C.F. (2015). Pharmacokinetics, biodistribution and cell uptake of antisense oligonucleotides. Adv. Drug Deliv. Rev..

[54] Juliano R.L. (2016). The delivery of therapeutic oligonucleotides. Nucleic Acids Res..

[55] Fluiter K., ten Asbroek A.L.M.A., de Wissel M.B., Jakobs M.E., Wissenbach M., Olsson H., Olsen O., Oerum H., Baas F. (2003). In vivo tumor growth inhibition and biodistribution studies of locked nucleic acid (LNA) antisense oligonucleotides. Nucleic Acids Res..

[56] Sun X.K., Fang H.F., Li X.X., Rossin R., Welch M.J., Taylor J.S. (2005). MicroPET imaging of MCF-7 tumors in mice via unr mRNA-targeted peptide nucleic acids. Bioconjugate Chem..

[57] Debart F., Abes S., Deglane G., Moulton H.M., Clair P., Gait M.J., Vasseur J.-J., Lebleu B. (2007). Chemical modifications to improve the cellular uptake of oligonucleotides. Curr. Top. Med. Chem..

[58] Stein C., Hansen J.B., Lai J., Wu S., Voskresenskiy A., H⊘ g A., Worm J., Hedtjärn M., Souleimanian N., Miller P. (2010). Efficient gene silencing by delivery of locked nucleic acid antisense oligonucleotides, unassisted by transfection reagents. Nucleic Acids Res..

[59] Hagedorn P.H., Persson R., Funder E.D., Albaek N., Diemer S.L., Hansen D.J., Moller M.R., Papargyri N., Christiansen H., Hansen B.R., Hansen H.F., Jensen M.A., Koch T. (2018). Locked nucleic acid: modality, diversity, and drug discovery. Drug Discov. Today.

[60] Jain R.K., Baxter L.T. (1988). Mechanisms of Heterogeneous distribution of monoclonal-antibodies and other macromolecules in tumors - significance of elevated interstitial pressure. Cancer Res..

[61] Minchinton A.I., Tannock I.F. (2006). Drug penetration in solid tumours. Nat. Rev. Cancer.

[62] Juliano R.L., Carver K. (2015). Cellular uptake and intracellular trafficking of oligonucleotides. Adv. Drug Deliv. Rev..

[63] Frazier K.S. (2015). Antisense oligonucleotide therapies: the promise and the challenges from a toxicologic pathologist's perspective. Toxicol. Pathol..

