# Supplementary material for: Cellular uptake, tissue penetration, biodistribution, and biosafety of threose nucleic acids: Assessing in vitro and in vivo delivery
Source: Mater Today Bio. 2022 May 18;15:100299. doi: 10.1016/j.mtbio.2022.100299 (PMC9142632; doi:10.1016/j.mtbio.2022.100299)
Supplement: Multimedia component 1 [file mmc1.docx]

Supplementary Materials

**Cellular Uptake, Tissue Penetration, Biodistribution, and Biosafety of Threose Nucleic Acids: Assessing In Vitro and In Vivo Delivery**

Fei Wang,^a^ Ling Sum Liu,^a^ Pan Li,^a^ Cia Hin Lau,^b^ Hoi Man Leung,^a^ Y Rebecca Chin,^c^ Chung Tin,^b,^ Pik Kwan Lo^a,d,^*

^a^ Department of Chemistry, City University of Hong Kong, Tat Chee Avenue, Kowloon Tong, Hong Kong SAR, China

^b^ Department of Biomedical Engineering, City University of Hong Kong, Tat Chee Avenue, Kowloon Tong, Hong Kong SAR, China

^c^ Tung Biomedical Sciences Centre, Department of Biomedical Sciences, City University of Hong Kong, Tat Chee Avenue, Kowloon Tong, Hong Kong SAR, China

^d^ Key Laboratory of Biochip Technology, Biotech and Health Care, Shenzhen Research Institute of City University of Hong Kong, Shenzhen 518057, China

* Corresponding author: Pik Kwan Lo. (Email: [peggylo@cityu.edu.hk](mailto:peggylo@cityu.edu.hk); Tel: +852 3442 7840; Fax: +852 3442 0552).

**1. Experimental Section**

**1.1 Materials and Reagents**

Tris(hydroxylmethyl)aminomethane, glycine, sodium dodecyl sulfate (SDS), ammonium persulfate (APS), N, N, N′, N′-Tetramethylethylenediamine (TEMED), dimethyl sulfoxide (DMSO), StainsAll, ammonium hydroxide, 3-(4,5-Dimethylthiazol-2-yl)-2,5-diphenyltetrazolium bromide (MTT), agarose, and Hoechst 33258 were purchased from Sigma-Aldrich. Fetal bovine serum (FBS), Dulbecco’s Modified Eagle Medium (DMEM), phosphate buffered saline (PBS), penicillin-streptomycin solution, and trypsin were bought from Invitrogen. 40 % Acrylamide/Bis-acrylamide solution (19:1) was obtained from Bio-Rad. Modified controlled pore glass (CPG, 1000 Å), 1-[(2-cyanoethyl)-(N, N-diisopropyl)]-phosphoramidite, and any other chemicals for TNA synthesis were purchased from BioAutomation. Sephadex G-25 was bought from Amersham Biosciences. All chemicals were used as received without any further purification unless indicated.

**1.2 Chemical Synthesis of TNA polymers**

TNA polymers were synthesized according to a reported solid-phase synthetic protocol which involves the standard cyanoethylphosphoramidite chemistry (Fig. S1) [1]. Four _L_-threofuranosyl nucloside monomers **3a-d** were synthesized from 1-*O*-Acetyl-2-*O*-benzoyl-3-*O*-tert-butyldiphenylsilyl-_L_-threofuranose **1**. Four different TNA nucleosides **2 a-d** were formed via the Silyl-Hilbert-Johnson reaction by reacting compound **1** with corresponding protected or unprotected nucleobases in the presence of a Lewis acid and then followed by removing the 3’-silyl protecting groups using tetrabutylammonium fluoride. The 3’-OH group on compound **2** was then protected with DMT functional group under basic media and then followed by deprotection of 2’-OH group using sodium hydroxide solution to generate **3a-d**. In our strategy, 2-cyanoethyl *N*,*N*,*N*',*N*'-tetraisopropylphosphoramidite was used to phosphorylate compounds **3a-d**, giving rise to the corresponding 2’-phosphoramidites TNA monomers **4a-d.** Sequence-defined TNA polymers were synthesized With the monomers **4a-d** on a controlled pore glass (CPG) solid support using an automated nucleic acid synthesizer.

**1.3 Half-life Calculation**.

To determine the mean half-life, bands at time point 0 were identified and used as baseline to remove the background intensity. For DNA, the decay rate (λ) and half-life (t_1/2_) were derived as follows.

Ι = Ι_𝑜_𝑒^-𝜆t^

𝜏 = $\frac{1}{\lambda}$

𝑡_1/2_ = 𝜏𝐼𝑛2 = 2.22 h

where τ is time constant, I*_0_* is the initial band intensity, and I is the band intensity at time t.

**2. Tables and Figures**

Table S1 Sequence and Mass of the TNA and DNA strands

| **Strand** | **Sequence** | **Calculated Mass (g/mol)** | **MALDI-TOF MS (g/mol)** |
| --- | --- | --- | --- |
| TNA-Cy3 | 3’-TCAGACTGATGTTGA-Cy3-2’ | 4888.09 | 4903.3164 |
| TNA-Cy5 | 3’-TATATATATCTCTCTCT-Cy5-2’ | 5363.40 | 5379.1348 |
| DNA-Cy3 | 5’-TCAGACTGATGTTGA-Cy3-3’ | 5114.67 | 5108.1445 |

**Fig. S1**. Synthetic scheme and structures of TNA polymer and its intermediates.


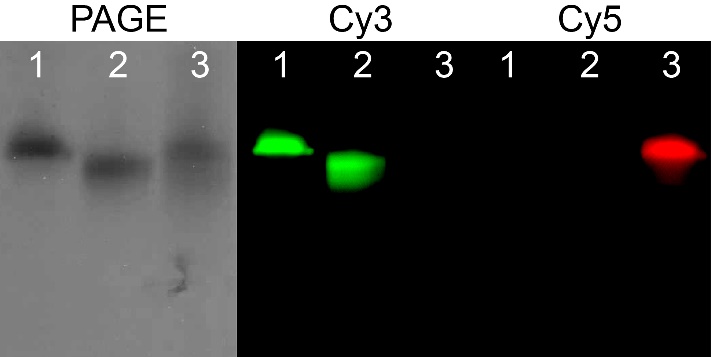


**Fig. S2.** Denaturing PAGE gel analysis of TNA and DNA strands and corresponding fluorescent images. Lane 1: TNA-Cy3 strands; Lane 2: DNA-Cy3 strands; Lane 3: TNA-Cy5 strands.


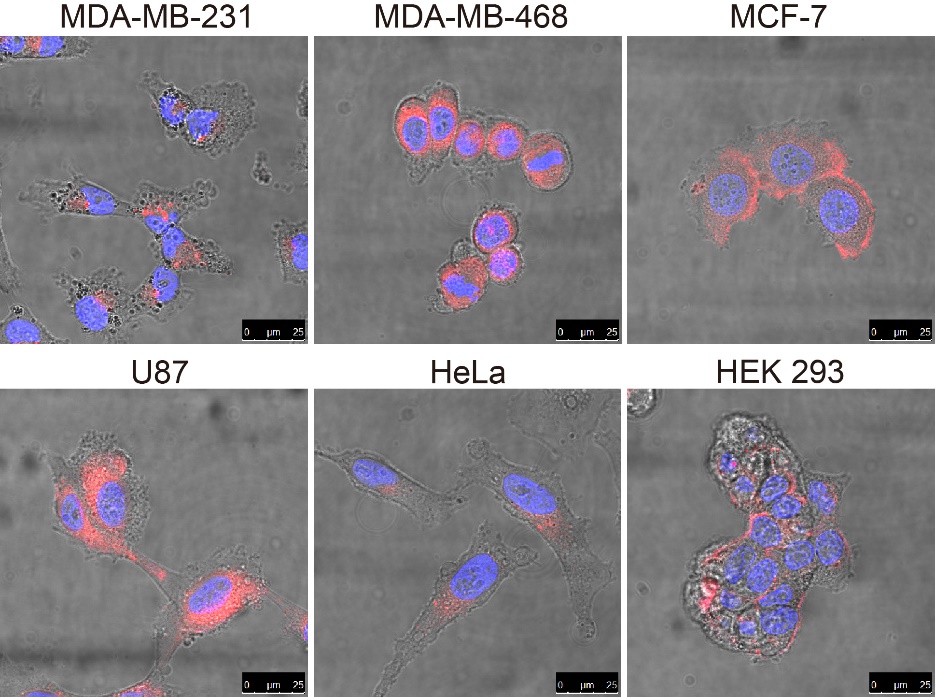


**Fig. S3.** CLSM images of various cell lines incubated with TNA-Cy3 oligonucleotides (0.2 μM) for 24 h. Nucleus was stained with Hoechst 33258 dye. Scale bar = 25 μm.


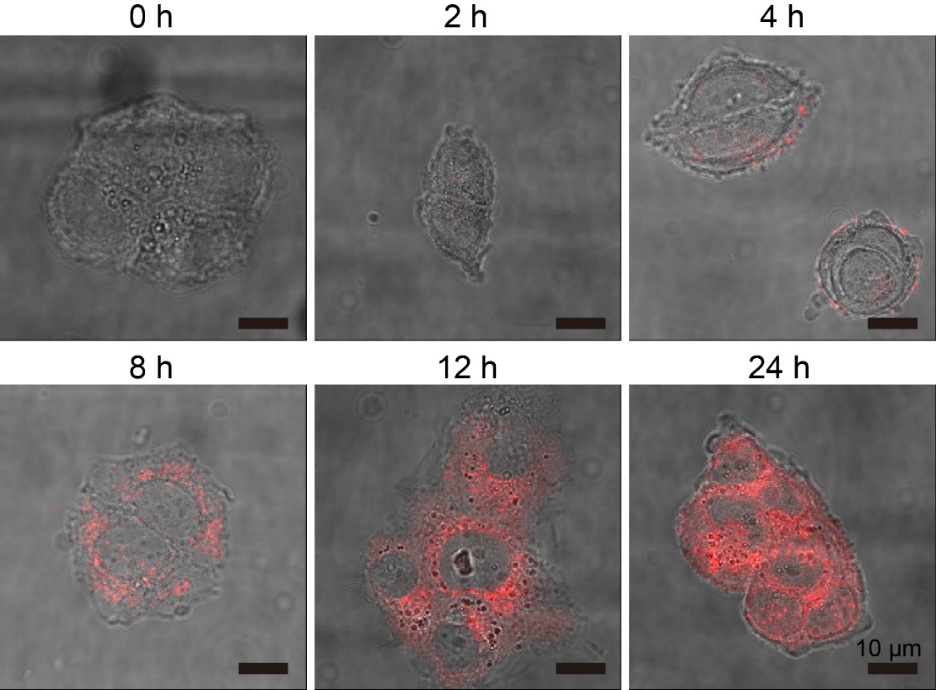


**Fig. S4.** CLSM images of MCF-7 cells incubated with TNA-Cy3 oligonucleotides (0.2 μM) for various time points (0, 2, 4, 8, 12, and 24 h). Scale bar = 10 μm.


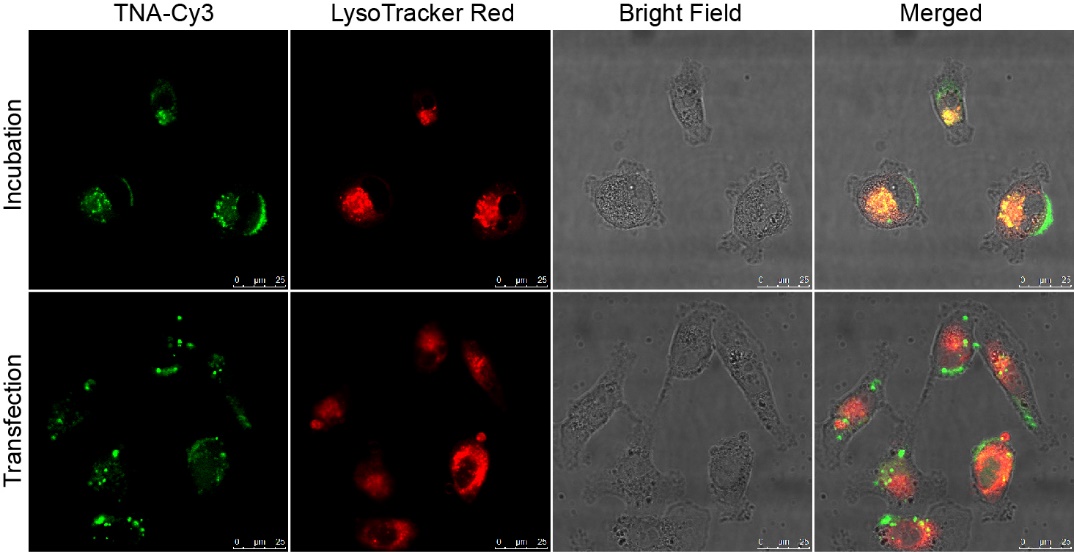


**Fig. S5.** CLSM images of MDA-MB-231 cells after TNA incubation/TNA transfection and then followed by LysoTracker Red staining.


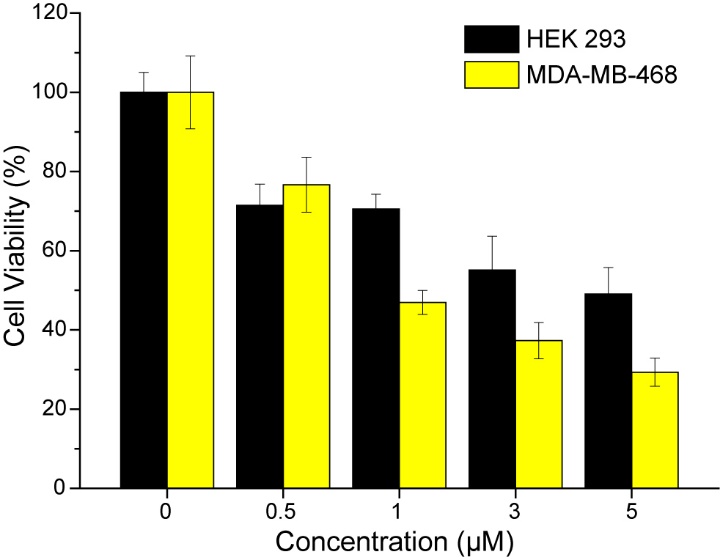


**Fig. S6.** Cytotoxicity assessment of chemotherapeutic drug Dox in HEK 293 and MDA-MB-468 cells via MTT assays. Data are shown as the mean ± SD (n = 6).


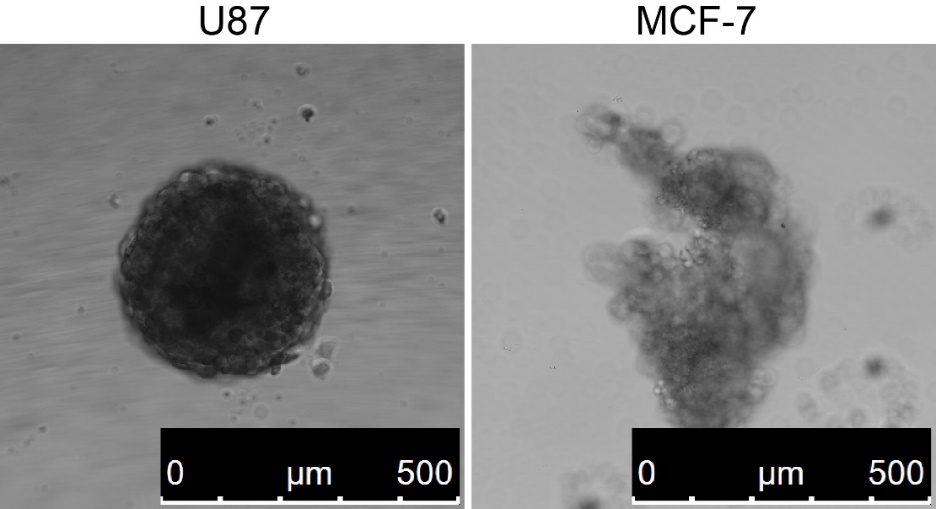


**Fig. S7.** Morphology of the U87 and MCF-7 3D multicellular spheroids. Scale bar = 500 μm.


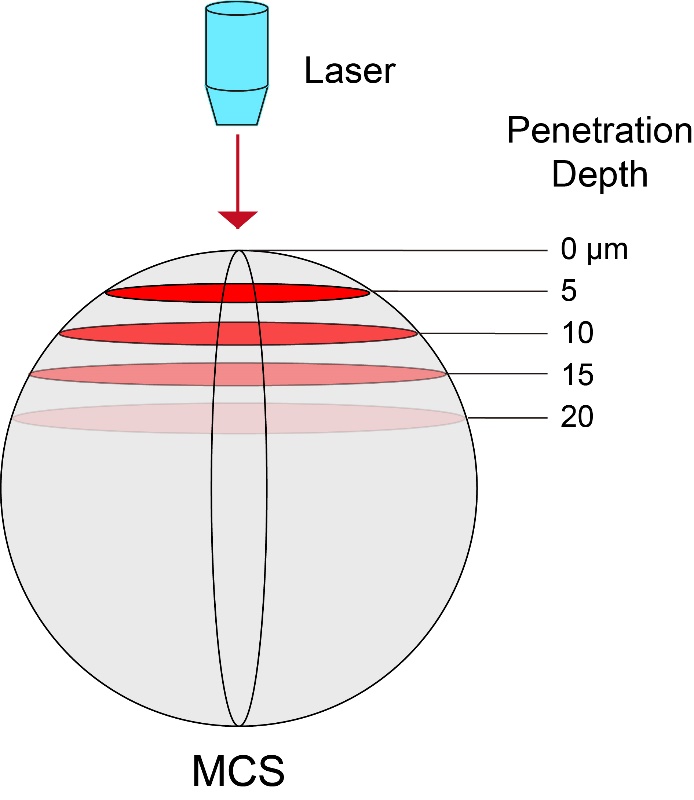


**Fig. S8.** Schematic illumination of determination of TNA penetration depth via CLSM analysis. The multicellular spheroids were imaged every 5 μm to determine the penetration depth. The 3D images were also obtained from the reconstruction of all confocal images.


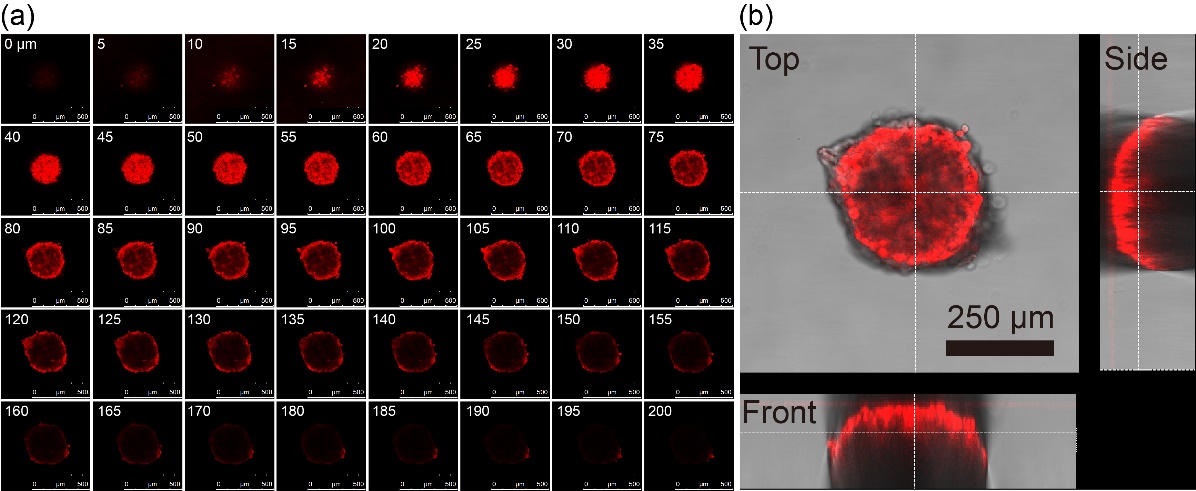


**Fig. S9.** (a) CLSM images of one example of U87 multicellular spheroid incubated with TNA-Cy3 oligonucleotides. The top left number represents the depth beneath the top surface. Scale bar = 500 μm. The penetration depth was determined to be 75 μm accordingly. (b) 3D image showing the top, side, and front view of the U87 multicellular spheroid reconstructed from the images shown in Fig. S7a. Scale bar = 250 μm.


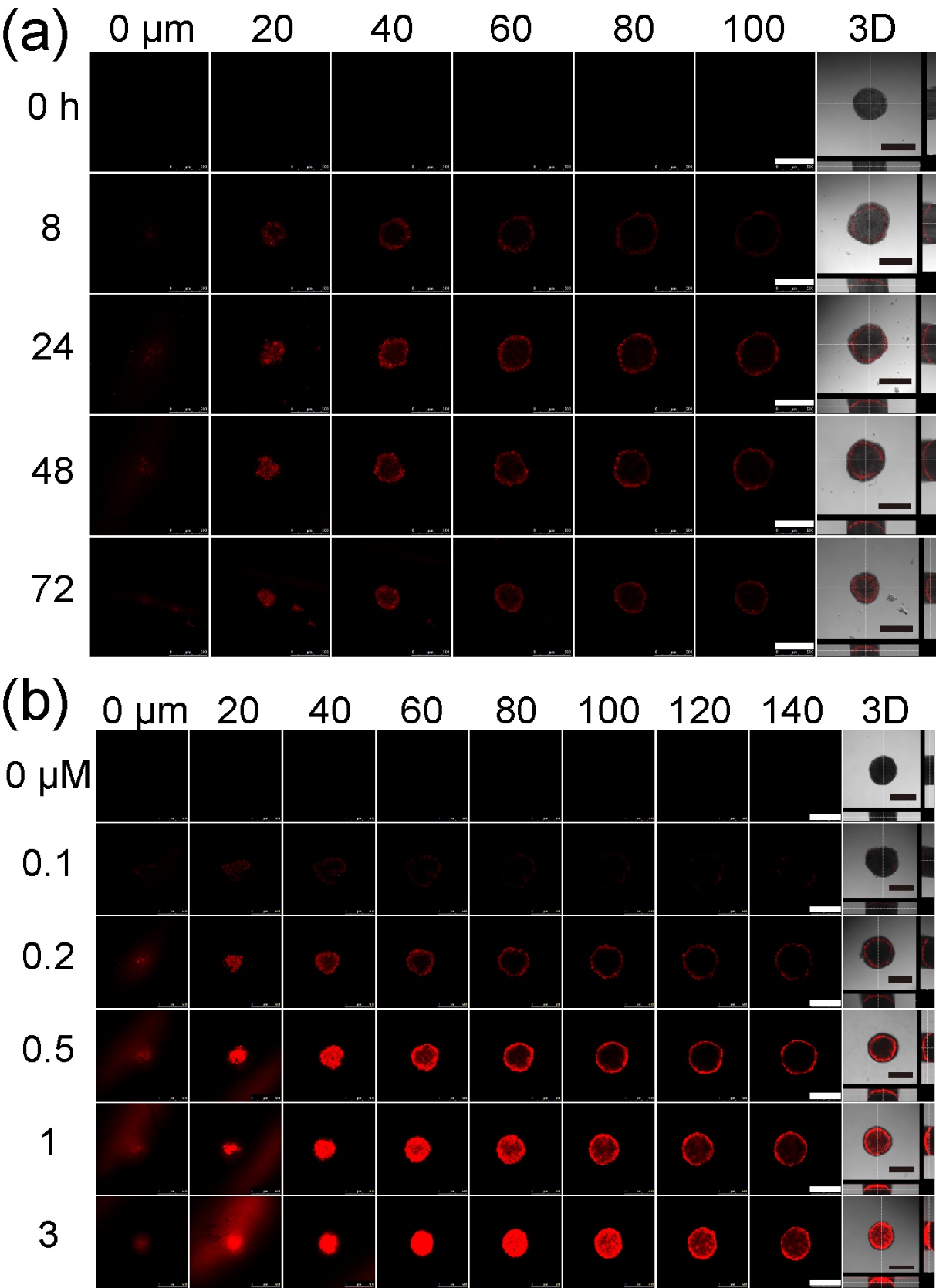


**Fig. S10.** (a) CLSM images of U87 multicellular spheroids incubated with TNA-Cy3 oligonucleotides (0.2 μM) for various time points (0, 8, 24, 48, and 72 h). Scale bar = 500 μm. (b) CLSM images of U87 multicellular spheroids incubated with TNA-Cy3 oligonucleotides of various concentrations (0, 0.1, 0.2, 0.5, 1, and 3 μM) for 48 h. Scale bar = 500 μm.


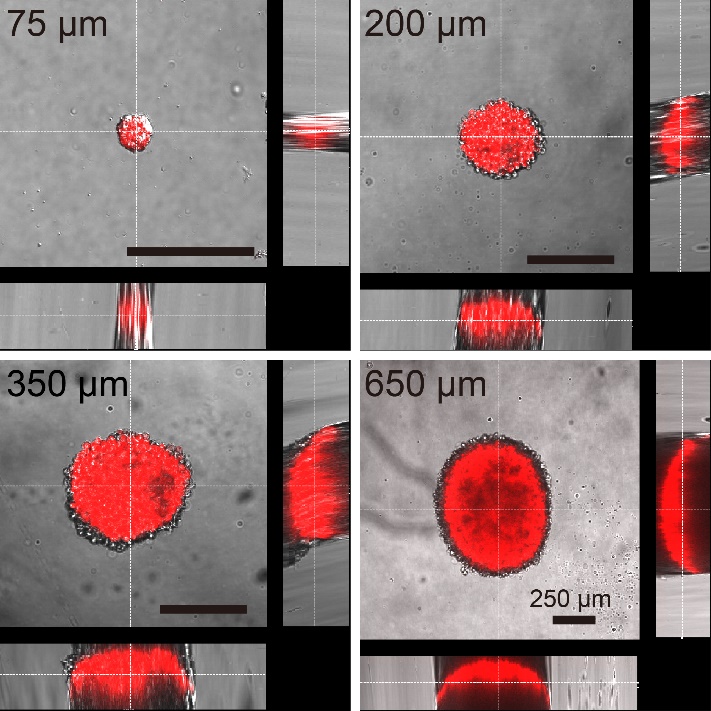


**Fig. S11.** 3D reconstruction images of U87 multicellular spheroids of various sizes incubated with TNA-Cy3 oligonucleotides for 48 h. The top left number represents the diameter of the multicellular spheroid. Scale bar = 250 μm.

References

[1] L.S. Liu, H.M. Leung, D.Y. Tam, T.W. Lo, S.W. Wong, P.K. Lo, alpha-L-Threose Nucleic Acids as Biocompatible Antisense Oligonucleotides for Suppressing Gene Expression in Living Cells, ACS Appl. Mater. Interfaces 10 (2018) 9736-9743.
